# Procalcitonin for diagnosing infected pancreatic necrosis: a systematic review and meta-analysis

**DOI:** 10.1186/s12876-026-05216-3

**Published:** 2026-08-13

**Authors:** Tianrong He, Yangfan Zhang, Xiaoling Wen, Zuopeng Zhang

**Affiliations:** 1 Department of Emergency, Zhongshan People’s Hospital, Zhongshan, 528400 China; 2 Department of Emergency, The First Affiliated Hospital of Guangzhou Medical University, Guangzhou, 510120 China

**Keywords:** Procalcitonin, Infected pancreatic necrosis, Acute pancreatitis, Diagnostic accuracy, Meta-analysis, Systematic review, GRADE evidence grading, Biomarker

## Abstract

**Background:**

Infected pancreatic necrosis (IPN) ranks among the most severe local complications of acute pancreatitis (AP) and plays a central role in driving AP-related death. Procalcitonin (PCT), a serum biomarker that responds preferentially to bacterial infection, has theoretical promise for differentiating infected from sterile necrosis. Earlier systematic reviews, though, are limited by small sample sizes, shallow subgroup analysis, and a lack of formal evidence grading. An updated synthesis that focuses specifically on IPN as the clinical endpoint is missing.

**Aim:**

This meta-analysis primarily aimed to evaluate the diagnostic performance of PCT for detecting IPN in patients with AP. Prediction of severe acute pancreatitis (SAP) was assessed as a secondary analysis, and evidence on PCT-guided antibiotic therapy was summarized as an exploratory analysis.

**Methods:**

PubMed, Embase, and the Cochrane Library were searched for original studies that assessed PCT for diagnosing IPN (primary objective), predicting severe AP (SAP; secondary analysis), or guiding antibiotic use in AP patients. Pooled sensitivity, specificity, likelihood ratios, and diagnostic odds ratios were calculated through a bivariate random-effects model. Hierarchical summary receiver operating characteristic (HSROC) curves were plotted. Pre-specified subgroup analyses covered PCT cutoff value, sampling time, study design, and reference standard. QUADAS-2 was used for bias assessment; evidence certainty was rated with the GRADE-DTA framework.

**Results:**

Thirty-nine studies met inclusion criteria: 16 on PCT for IPN diagnosis (2,156 patients; 512 IPN cases), 20 on PCT for SAP prediction (3,386 patients; 934 SAP cases), and 3 randomized controlled trials on PCT-guided antibiotic therapy. For IPN diagnosis, pooled sensitivity was 0.82 (95% CI: 0.75–0.87), pooled specificity 0.84 (95% CI: 0.78–0.89), and area under the curve (AUC) 0.90 (95% CI: 0.87–0.92); GRADE certainty was low. For SAP prediction, pooled sensitivity was 0.83, specificity 0.76, AUC 0.87; evidence certainty was low. A cutoff of 1.0-3.5 ng/mL gave the best trade-off between sensitivity and specificity (AUC 0.92). Serial dynamic monitoring (AUC 0.93) outperformed single admission measurement (AUC 0.85) by a wide margin.

**Conclusions:**

PCT shows good overall ability to identify IPN and may be incorporated as an adjunctive biomarker for IPN risk stratification. A cutoff range of 1.0–3.5 ng/mL and serial monitoring may help clinical risk stratification. These cutoff-related findings require external validation in prospective multicenter studies with pre-specified cutoffs and standardized adjudicated reference criteria integrating clinical, radiologic, microbiologic, and procedural evidence, with culture confirmation incorporated when drainage or necrosectomy is clinically performed.

**Supplementary Information:**

The online version contains supplementary material available at https://doi.org/10.1186/s12876-026-05216-3.

## Introduction

Acute pancreatitis (AP) is one of the most common gastrointestinal emergencies. Its global incidence has been climbing for decades. A systematic review pooling multinational epidemiological data reported that the annual incidence rose from roughly 20 per 100,000 in the late 20th century to over 34 per 100,000 in recent years [1]. Most AP patients run a self-limiting interstitial edematous course, but about 20%–30% progress to necrotizing pancreatitis. That proportion fluctuates across regions depending on healthcare resources; low- and middle-income countries bear disproportionately higher rates of necrotic transformation and death [2]. The 2012 Revised Atlanta Classification splits necrotizing pancreatitis into sterile necrosis and infected pancreatic necrosis (IPN) based on whether bacteria have colonized the necrotic tissue. These two subtypes carry very different prognoses, and the classification still serves as the backbone for clinical stratification [3]. IPN matters because it shapes AP outcomes in a decisive way. A prospective multicenter study identified organ failure and IPN as the two independent determinants of AP mortality; when both are present, the death rate can exceed 30% [4]. A meta-analysis of necrotizing pancreatitis confirmed that patients with IPN die at significantly higher rates than those with sterile necrosis, making infection status an independent adverse prognostic factor [5]. AP outcomes are also influenced by metabolic-endocrine comorbidities. Recent work shows that individual components of the metabolic syndrome can independently worsen AP prognosis, pointing to a real role for endocrine-metabolic disturbances in the pancreatic inflammatory cascade [6]. Taken together, AP is a disease whose incidence keeps growing and whose clinical behavior is highly heterogeneous. IPN stands out as its most severe local complication and as the key decision point for whether to start interventional treatment [7].

Early and accurate diagnosis of IPN remains clinically challenging. Ultrasound- or CT-guided fine-needle aspiration (FNA) with microbiological culture has long been treated as the reference standard. The technique has real drawbacks, though. It is invasive and carries procedure-related risks. Its false-negative rate runs between 12% and 25%, partly because infected foci within necrotic tissue are unevenly distributed and a single needle pass may miss them [8]. The routine use of FNA has come under question as evidence accumulates. One retrospective cohort compared a routine FNA strategy with a selective strategy triggered only by clinical indications and found no meaningful difference in IPN detection rate or patient outcomes, suggesting FNA’s diagnostic yield may not justify routine use [9]. On the imaging side, gas bubbles within necrotic areas on contrast-enhanced CT are highly specific for IPN but not very sensitive-visible in only about 30%–40% of IPN cases—and they tend to appear after infection has already set in. At the same time, AP patients, especially those with necrotizing pancreatitis, often show signs of systemic inflammatory response syndrome (SIRS) early on: fever, elevated white cell counts, hemodynamic shifts. These non-specific features overlap heavily with IPN, making it unreliable to distinguish sterile SIRS from genuine bacterial infection on clinical grounds alone. All of this underscores the urgent need for non-invasive serum biomarkers that can separate the two.

Procalcitonin (PCT) is a 116-amino-acid precursor of calcitonin whose molecular properties give it a built-in advantage for telling infection apart from sterile inflammation. Under normal conditions, thyroid C cells produce PCT through tissue-specific CALC-1 gene transcription and post-translational processing, then cleave it into mature calcitonin for calcium-phosphorus regulation. Circulating PCT stays very low (< 0.05 ng/mL) [10]. During bacterial infection and sepsis, the CALC-1 expression pattern changes fundamentally. Non-neuroendocrine tissues—lung, liver, kidney, intestinal epithelium-begin synthesizing PCT in response to bacterial endotoxins and pro-inflammatory cytokines like IL-1β, TNF-α, and IL-6 [11]. The same ectopic expression has been documented in human adipose tissue; adipocytes in an inflammatory milieu can become a meaningful source of PCT, broadening what we know about PCT production and hinting at deeper connections between PCT synthesis and metabolic-endocrine regulation [12]. These non-thyroidal cells lack the enzymes needed to process PCT into mature calcitonin. The intact precursor accumulates in the blood and can rise several hundredfold within 4–6 h after infection begins. Viral infections and sterile inflammation trigger much smaller increases. That differential response is what gives PCT its biological basis for separating bacterial infection from non-infectious inflammation. Applied to AP, PCT should be able to distinguish IPN-where bacteria have invaded-from sterile SIRS driven by inflammatory mediators, offering clinicians a critical piece of diagnostic information.

Several systematic reviews and meta-analyses have looked at PCT’s diagnostic and predictive value in AP. Early reviews tentatively confirmed some predictive ability for AP severity and IPN, but the pool of available primary studies was small and the methodological frameworks limited, so the conclusions lacked precision and clinical actionability [13]. More recent analyses have focused mostly on predicting AP severity overall or on comparing PCT head-to-head with C-reactive protein (CRP), tilting the research lens toward general AP prognosis rather than IPN as a distinct endpoint. A wave of new primary studies has appeared since 2017-prospective cohorts from different countries and healthcare systems-and none of it has been pulled together systematically. On the methods side, gaps are clear: no meta-analysis has stratified by PCT cutoff value, sampling time window, or assay method, all of which are major drivers of between-study heterogeneity. No one has graded the evidence certainty for PCT in diagnosing IPN using the GRADE-DTA framework. These gaps motivated the current work. The primary objective of this meta-analysis was to evaluate the diagnostic accuracy of serum PCT for IPN as a distinct clinical endpoint in patients with AP, using a bivariate random-effects model, predefined subgroup analyses, and GRADE-DTA evidence assessment. Because PCT is also used in broader AP severity assessment and infection-management decisions, SAP prediction was examined as a secondary analysis, and PCT-guided antibiotic therapy was summarized separately as an exploratory antibiotic-stewardship analysis.

## Materials and methods

### Reporting standards

Reporting strictly follows the latest requirements for standardized reporting of systematic reviews set forth in the PRISMA 2020 statement [14]. Given that diagnostic accuracy is the core analytical focus, the PRISMA-DTA extension for systematic reviews of diagnostic test accuracy was additionally followed [15].

### Inclusion and exclusion criteria

Criteria were built on the PICOS framework. Adults (≥ 18 years) with clinically and/or radiologically confirmed AP were eligible regardless of severity or etiology.

Studies had to evaluate serum or plasma PCT as the index test; assay method and cutoff were left unrestricted to keep the analysis broad and to feed subgroup analyses later. For IPN-targeted studies, accepted reference standards were FNA with positive microbiological culture, positive intraoperative tissue culture, or a composite judgment combining gas bubbles on contrast-enhanced CT with clinical infection evidence. For AP severity studies, grading had to follow the Revised Atlanta Classification or another recognized severity scoring system. Studies needed to report enough data to build a 2 × 2 contingency table (TP, FP, FN, TN) or to allow direct calculation of sensitivity and specificity. IPN diagnosis was designated as the primary analytical objective; SAP prediction was treated as a secondary analysis to provide a comparative reference for PCT’s diagnostic profile across different clinical endpoints. Prospective and retrospective observational designs were included, along with RCTs that reported PCT diagnostic data.

Studies were excluded if they dealt only with chronic pancreatitis, post-surgical pancreatic complications, or non-pancreatic intra-abdominal infections. Also excluded: studies reporting PCT only as a continuous variable without classification data; studies embedding PCT in a composite model without separate performance figures; animal or in vitro experiments; reviews; data-incomplete conference abstracts; case reports; and expert opinion pieces. When publications overlapped, the version with the largest sample or most complete data was kept. Language was limited to English and Chinese.

### Search strategy

The search was completed on November 15, 2025 and covered PubMed/MEDLINE, Embase, and the Cochrane Library (including the Cochrane Database of Systematic Reviews and the Central Register of Controlled Trials) from inception onward. This study was designed to focus on major internationally indexed biomedical databases with broad coverage, controlled indexing, and reproducible search records. No language restriction was applied. Chinese-language studies were eligible if identified through the searched databases, reference-list checking, or forward citation tracking. Two concept modules drove the strategy: Module 1 captured procalcitonin and its spelling variants using both subject headings and free-text terms; Module 2 captured acute pancreatitis, necrotizing pancreatitis, infected pancreatic necrosis, peripancreatic necrosis, and walled-off necrosis the same way. Terms within each module were linked with OR; the two modules were crossed with AND. Syntax was adapted for each database; the full strategy appears in the supplementary materials. Reference lists of all included studies and relevant systematic reviews were hand-checked. Forward citation tracking through Google Scholar was done for key papers to minimize missed records.

### Study selection and data extraction

Selection followed a two-stage dual-reviewer process. Two reviewers independently screened titles and abstracts of all deduplicated records, then independently assessed the full texts of articles marked “potentially eligible” or flagged with disagreement. Conflicts at both stages were resolved by discussion; a third reviewer arbitrated when needed. Agreement was measured with Cohen’s kappa. The whole process was logged per the PRISMA flow diagram.

Two reviewers independently extracted data into a pilot-tested standardized spreadsheet and cross-checked results. Fields covered study characteristics (authors, year, country, design, single-/multi-center), patient characteristics (sample size, demographics, AP severity distribution, IPN/SAP rates), PCT details (assay method, sampling time, cutoff value, how it was chosen), reference standard definitions, and 2 × 2 table data. The full extraction template is in the Table S2. When a study gave sensitivity, specificity, and sample size but not the raw four-cell counts, absolute values were back-calculated. If a study reported more than one cutoff, the primary analysis used whichever the authors labeled optimal or whichever is most widely used in the field; all cutoffs fed into subgroup analyses.

### Quality assessment

Diagnostic accuracy studies in Groups A and B were appraised with QUADAS-2 [16], covering patient selection, index test, reference standard, and flow and timing. Each domain was judged for both bias risk and applicability concerns, rated “low risk,” “high risk,” or “unclear.” Domain-specific signaling questions were tailored to the review’s context beforehand (Table S3). Group C RCTs were assessed with the Cochrane Risk of Bias 2.0 tool. Two reviewers worked independently; disagreements were settled by discussion or a third reviewer.

### Statistical analysis

Sensitivity and specificity are inherently negatively correlated in diagnostic studies, so a bivariate random-effects model served as the primary method. It jointly models both parameters and produces pooled sensitivity, specificity, positive and negative likelihood ratios, and diagnostic odds ratio, each with 95% confidence intervals [17]. HSROC curves were drawn to show PCT’s diagnostic profile across thresholds, and AUC was computed as a global discrimination measure [18]. The primary analysis ran on Group A (IPN diagnosis); Group B (SAP prediction) was analyzed separately with the same approach.

To test whether diagnostic performance differed across PCT cutoff categories, we performed random-effects meta-regression using the study-level log diagnostic odds ratio as the dependent variable and cutoff group as the moderator. The variance of log diagnostic odds ratio was calculated from the extracted 2 × 2 tables. A continuity correction of 0.5 was applied when any cell contained zero. The global P value for the moderator was used as the formal interaction test for cutoff subgroup differences. As supplementary analyses, we also performed random-effects meta-regression using logit sensitivity and logit specificity as dependent variables. These interaction analyses were used to test between-study subgroup differences and did not replace the primary bivariate random-effects/HSROC model.

Heterogeneity was tested with Cochran’s Q (significant at *P* < 0.10) and I2 (> 75% = high) [19]. Threshold effect was checked with the Spearman correlation between log-sensitivity and log (1-specificity). Pre-planned subgroup analyses split by PCT cutoff (≤ 1.0, 1.0–3.5, > 3.5 ng/mL), sampling time, assay method, study design, IPN reference standard, and publication year (pre- vs. post-2012 Atlanta revision); each subgroup got its own bivariate model fit. Robustness was probed three ways: dropping high-bias-risk studies, dropping studies with *n* < 30, and leave-one-out. Publication bias was assessed with Deeks’ funnel plot asymmetry test [20].

Evidence certainty was graded per GRADE-DTA, starting from the study-design baseline and considering five possible reasons to downgrade: bias risk, indirectness, inconsistency, imprecision, and publication bias [21]. Upgrade factors were also weighed. Final certainty fell into one of four levels: high, moderate, low, or very low [22]. Meta-analyses were performed using Stata 17.0 (midas and metandi modules), whereas HSROC model fitting and related sensitivity analyses were conducted using SAS 9.4. The HSROC area under the curve (AUC) and corresponding 95% confidence intervals were derived from the fitted HSROC model parameters and their variance-covariance estimates. All *P* values are two-sided; *P* < 0.05 denotes statistical significance except for heterogeneity and publication bias tests (*P* < 0.10).

## Results

### Literature screening and characteristics of included studies

**Fig. 1:**
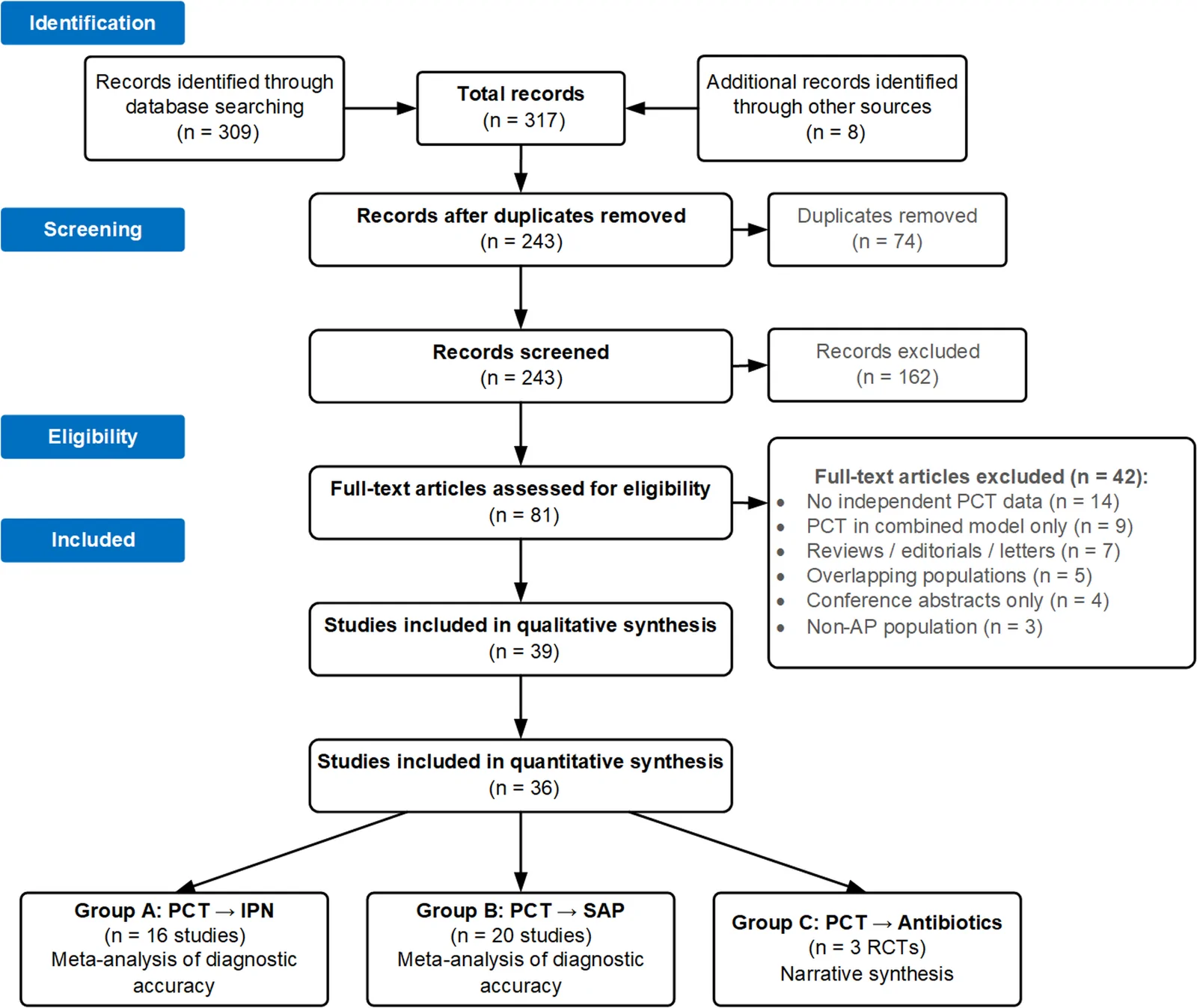
PRISMA flow diagram of study selection process

The database search returned 309 records. Reference screening and forward citation tracking added 8 more, giving 317 in total. Removing 74 duplicates left 243 for title-and-abstract review. Of those, 162 clearly fell outside the inclusion criteria and were dropped. The remaining 81 went to full-text assessment. Forty-two were excluded at that stage: 14 because independent PCT diagnostic classification data could not be extracted, 9 because PCT was only part of a composite model with no separate performance data, 7 because they were reviews, commentaries, or letters, 5 for overlapping populations, 4 for being conference abstracts without complete data, and 3 for studying non-AP populations. Cohen’s kappa between screeners was 0.87 at the title-abstract stage and 0.91 at full text, both reflecting high agreement. Thirty-nine studies made the final cut; the screening flow appears in Fig. 1.

Publication years spanned 1997 to 2025 across 20 countries and regions. China had the most papers (*n* = 13), followed by India (*n* = 4), Germany (*n* = 2, plus involvement in 1 multicenter study), Hungary (*n* = 2), Turkey (*n* = 2), and the United Kingdom (*n* = 2). One study each came from Finland, Switzerland, France, Spain, Denmark, Slovakia, Serbia, Sweden, Nepal, South Korea, Romania, Mexico, and the United States. Study 7 was an international multicenter effort spanning Germany, Finland, Italy, and Switzerland. Sample sizes ranged from 24 to 516. The 39 studies were sorted into three groups by their clinical endpoint: Group A—16 studies on PCT for IPN diagnosis (2,156 patients, 512 IPN cases); Group B—20 studies on PCT for SAP prediction (3,386 patients, 934 SAP cases); Group C—3 RCTs on PCT-guided antibiotic therapy. In Group A, 10 studies were prospective and 6 retrospective. In Group B, 11 were prospective (1 a registered cohort), 7 retrospective, 1 cross-sectional, and 1 observational. PCT assays included electrochemiluminescence immunoassay (ECLIA), enzyme-linked immunosorbent assay (ELISA), immunoluminometric assay, and the semi-quantitative PCT-Q rapid test. Only 1 Group A study used the semi-quantitative PCT-Q (Study 6); the rest were quantitative, which made assay-method subgroup analysis impractical in Group A. Baseline characteristics are in Table S1.

### Quality assessment

**Fig. 2:**
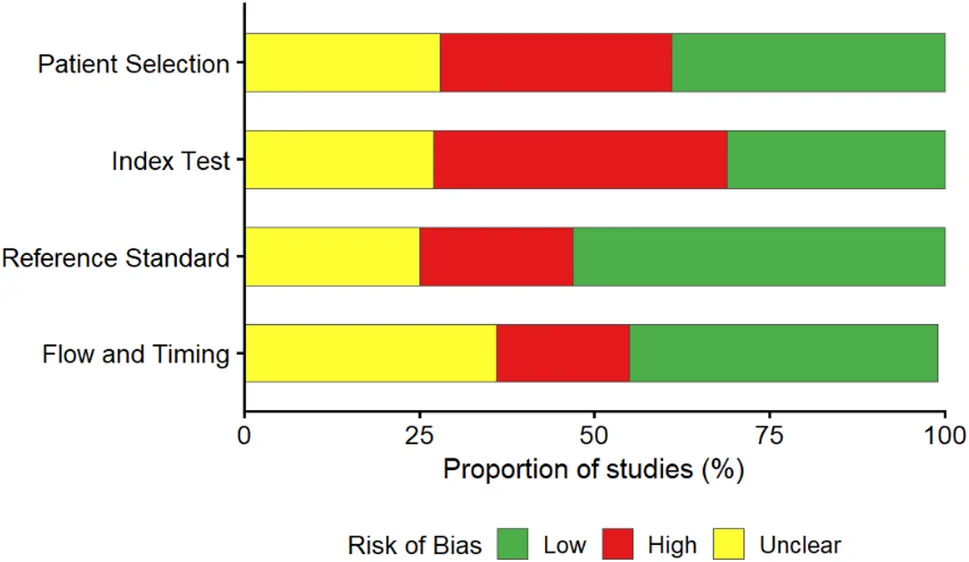
Summary of risk of bias assessment using the QUADAS-2 tool across included studies

**Fig. 3:**
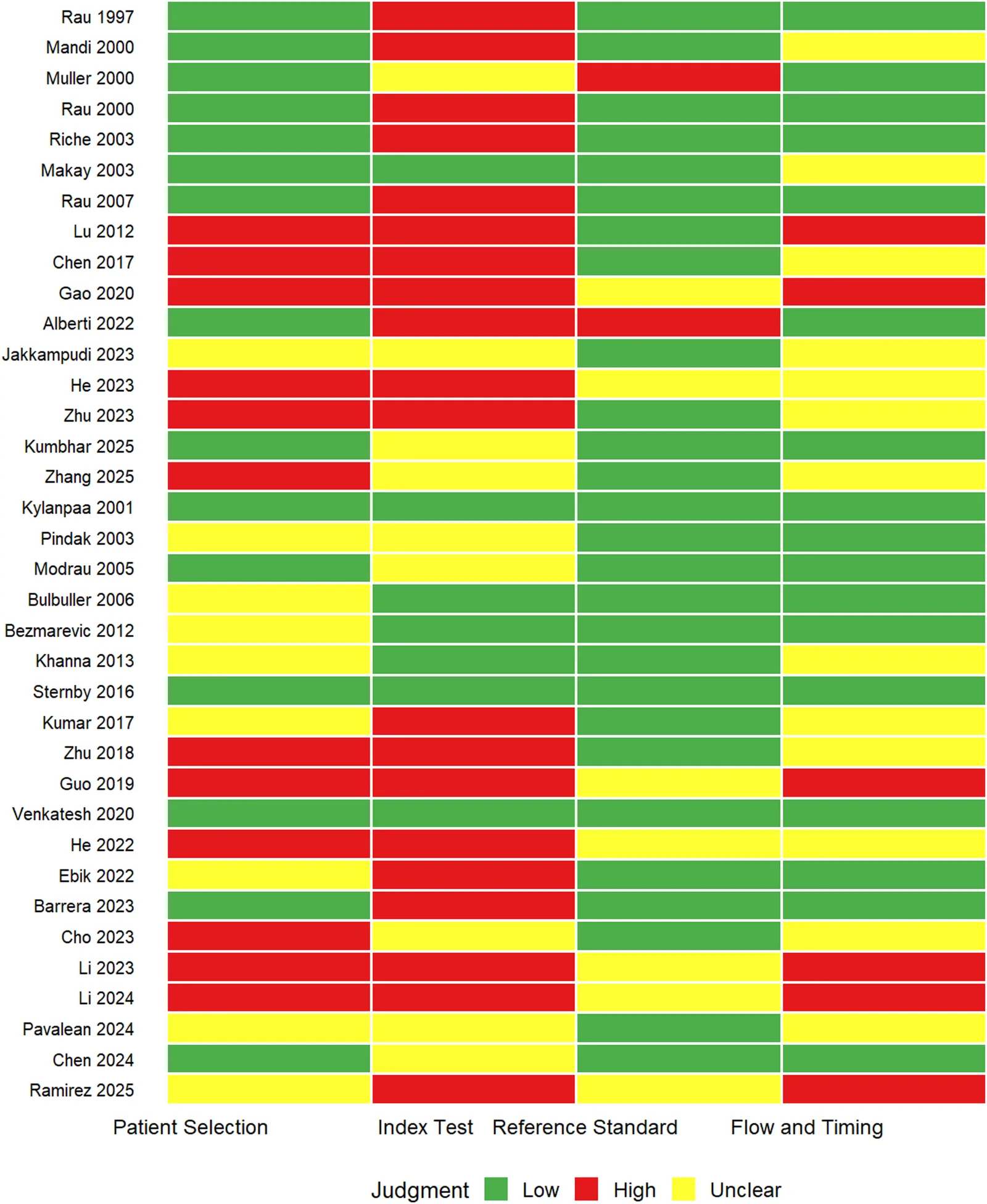
Risk of bias and applicability concerns for individual studies assessed by the QUADAS-2 tool

QUADAS-2 was applied to all 36 diagnostic accuracy studies across Groups A and B. Domain-level distributions appear in Fig. 2; study-by-study judgments are in Fig. 3. For patient selection, 14 studies (39%) scored low risk—consecutive enrollment or prospective cohort without inappropriate exclusions. Twelve (33%) scored high risk, mostly because of convenience sampling in retrospective designs or restriction to certain severity subgroups. Ten (28%) were unclear from poor reporting. The index test domain showed more problems: 15 studies (42%) scored high risk because the PCT cutoff was chosen after the fact by optimizing the ROC curve on the study’s own data, which can inflate performance estimates. Eleven (31%) had a pre-set cutoff or used a previously recommended threshold and scored low risk. Ten (27%) gave too little information to judge. In the reference standard domain, 19 studies (53%) used FNA with culture or intraoperative culture and scored low risk. Eight (22%) relied on imaging or composite clinical judgment without microbiological proof and scored high risk. Nine (25%) were unclear. For flow and timing, 16 studies (44%) scored low risk—all patients received the reference standard and the gap between PCT testing and outcome ascertainment was reasonable. Seven (19%) had partial verification bias or too long an interval and scored high risk. Thirteen (36%) reported too little to tell. On applicability, most studies raised low or unclear concerns for patient selection and the index test. The reference standard domain drew more concern because IPN criteria differed across studies (FNA culture vs. imaging vs. clinical judgment). The 3 Group C RCTs were assessed with Cochrane Risk of Bias 2.0. All showed low risk for randomization and outcome measurement but flagged some concerns for deviations from intended interventions, since clinical decisions could not always follow the PCT algorithm.

### Diagnostic accuracy of PCT for IPN

**Fig. 4:**
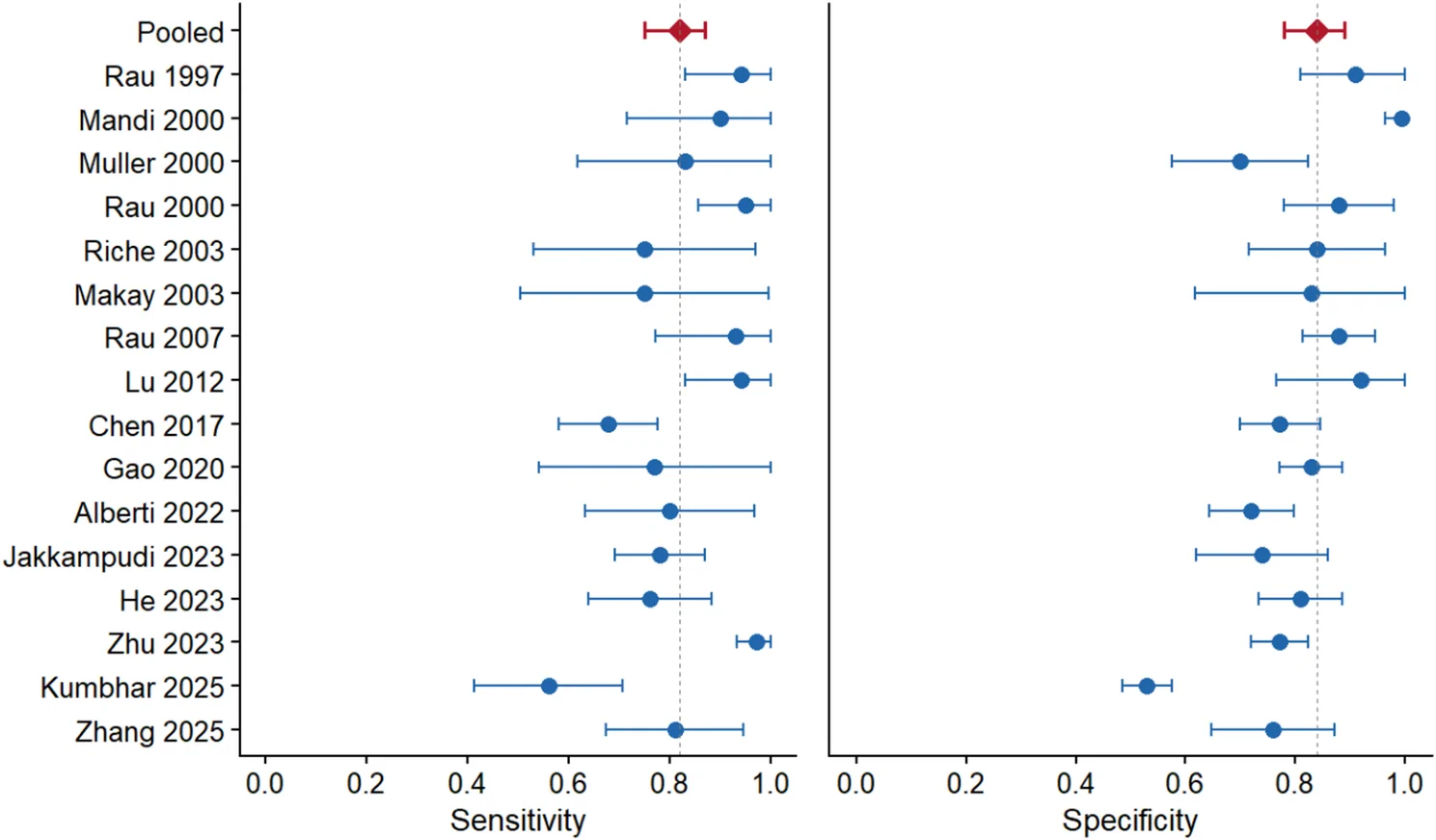
Coupled forest plots of pooled sensitivity and specificity of procalcitonin for diagnosing infected pancreatic necrosis

The bivariate random-effects model across the 16 Group A studies gave a pooled sensitivity of 0.82 (95% CI: 0.75–0.87), pooled specificity of 0.84 (95% CI: 0.78–0.89), pooled positive likelihood ratio of 5.18 (95% CI: 3.62–7.41), pooled negative likelihood ratio of 0.22 (95% CI: 0.15–0.31), and pooled diagnostic odds ratio of 23.8 (95% CI: 12.6–44.9). The paired forest plot is in Fig. 4.

**Fig. 5:**
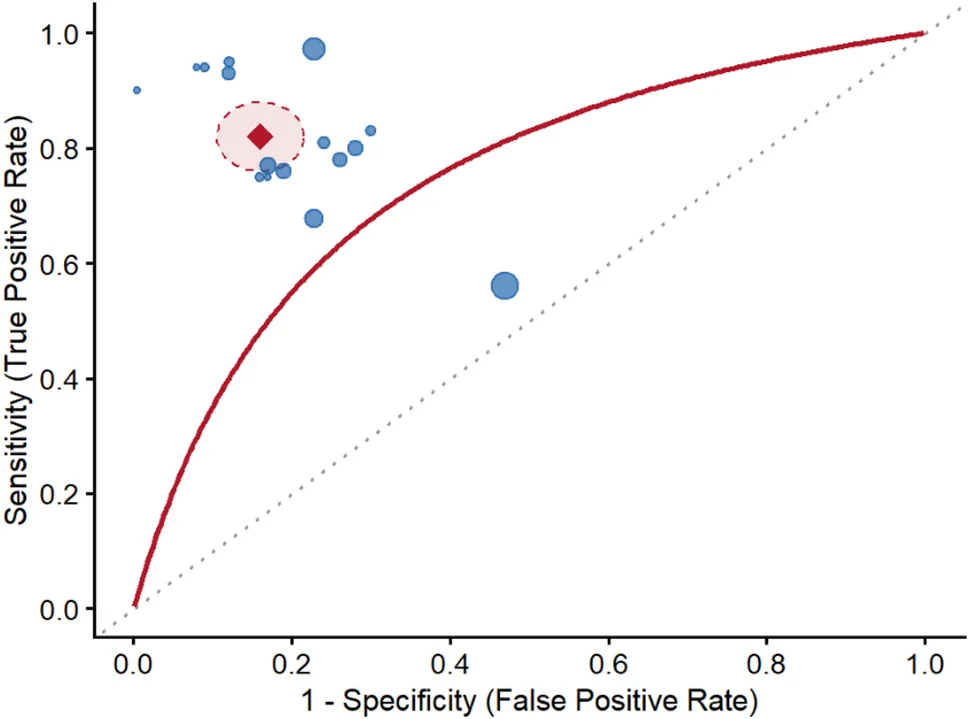
Hierarchical summary receiver operating characteristic (HSROC) curve of procalcitonin for diagnosing infected pancreatic necrosis

The HSROC curve gave an AUC of 0.90 (95% CI: 0.87–0.92), shown in Fig. 5, pointing to good overall discrimination. Between-study heterogeneity was high: I² was 82.4% for sensitivity (Cochran’s Q *P* < 0.001) and 79.6% for specificity (*P* < 0.001). The Spearman correlation was 0.32 (*P* = 0.228), not significant, meaning the threshold effect was not the main heterogeneity driver. Clinical and methodological differences across studies were the more likely explanation.

Subgroup analyses followed pre-specified variables; pooled results are in Table 1. Stratifying by cutoff: the 5 studies at ≤ 1.0 ng/mL had pooled sensitivity 0.73 (95% CI: 0.62–0.82), specificity 0.76 (95% CI: 0.66–0.84), AUC 0.81. The 10 studies at > 1.0 to < 3.5 ng/mL had pooled sensitivity 0.85 (95% CI: 0.78–0.90), specificity 0.86 (95% CI: 0.80–0.91), AUC 0.92. Only 1 study used ≥ 3.5 ng/mL (Study 7, cutoff 3.5 ng/mL)—too few for pooling—but it was a multicenter prospective study and individually reported 93% sensitivity and 88% specificity. For the hypothesis-generating PCT of 1.0-3.5 ng/mL, the pooled sensitivity and specificity were 0.85 and 0.86, respectively, corresponding to a positive likelihood ratio of 6.07 and a negative likelihood ratio of 0.17. Across assumed IPN prevalences of 10% to 50%, a positive PCT result increased the post-test probability of IPN from 40.3% to 85.9%, whereas a negative result reduced the post-test probability to 1.9% to 14.9% (Table S4). The pattern points to the moderate cutoff range as the hypothesis-generating risk-stratification interval between sensitivity and specificity. Stratifying by sampling time: 7 studies using a single draw at or within 72 h of admission had pooled sensitivity 0.78 (95% CI: 0.68–0.85), specificity 0.79 (95% CI: 0.71–0.86), AUC 0.85. The 7 studies that tracked PCT daily or at multiple time points had pooled sensitivity 0.87 (95% CI: 0.79–0.92), specificity 0.88 (95% CI: 0.81–0.93), AUC 0.93—suggesting serial monitoring beats a single reading. Two studies were left out of this split because of unusual clinical contexts: Study 8 measured PCT in FNA aspirate fluid, and Study 10 drew blood at drain removal. Stratifying by design: the 10 prospective studies had slightly better pooled sensitivity (0.84) and specificity (0.87) than the 6 retrospective ones (0.79 and 0.79), with AUCs of 0.92 vs. 0.86, consistent with selection bias in retrospective work pulling estimates down. Stratifying by reference standard: the 10 studies using FNA/culture had an AUC of 0.92 versus 0.84 for the 6 using clinical/imaging criteria, a gap likely tied to misclassification from unstandardized reference methods. Stratifying by publication year: the 7 pre-2012 Atlanta studies had a pooled AUC of 0.93; the 9 post-revision studies, 0.86. More recent studies tend to be more conservative in sample selection and cutoff choice, and the redefined Atlanta criteria may have shifted the makeup of enrolled populations. Earlier studies also tended to be small (median around 50 patients), and small diagnostic studies are known to overestimate performance—another factor that may have inflated pre-Atlanta era results.

**Table 1:** Subgroup analyses of the diagnostic accuracy of procalcitonin for infected pancreatic necrosis

Subgroup	No. of studies	Pooled Se (95% CI)	Pooled Sp (95% CI)	AUC	DOR (95% CI)
Overall	16	0.82 (0.75–0.87)	0.84 (0.78–0.89)	0.90 (0.86–0.94)	23.8 (12.6–44.9)
By PCT cutoff					
≤ 1.0 ng/mL	5	0.73 (0.62–0.82)	0.76 (0.66–0.84)	0.81 (0.78–0.84)	8.5 (4.2–17.3)
> 1.0–<3.5 ng/mL	10	0.85 (0.78–0.90)	0.86 (0.80–0.91)	0.92 (0.87–0.95)	33.1 (15.8–69.4)
≥ 3.5 ng/mL	1	—	—	—	—
By sampling time					
Admission / early single (≤ 72 h)	7	0.78 (0.68–0.85)	0.79 (0.71–0.86)	0.85 (0.83–0.89)	13.2 (6.4–27.1)
Serial / dynamic monitoring	9	0.87 (0.79–0.92)	0.88 (0.81–0.93)	0.93 (0.90–0.96)	46.3 (17.8–120.5)
By study design					
Prospective	10	0.84 (0.76–0.90)	0.87 (0.80–0.92)	0.92 (0.89–0.94)	32.4 (14.5–72.3)
Retrospective	6	0.79 (0.69–0.86)	0.79 (0.70–0.86)	0.86 (0.83–0.90)	14.1 (6.8–29.5)
By reference standard					
FNA / culture	10	0.84 (0.77–0.90)	0.87 (0.80–0.92)	0.92 (0.86–0.97)	33.8 (15.2–75.1)
Clinical / imaging	6	0.78 (0.66–0.87)	0.78 (0.68–0.86)	0.84 (0.81–0.88)	12.5 (5.6–27.9)
By publication year					
Before 2012 (pre-Atlanta revision)	7	0.86 (0.77–0.92)	0.88 (0.80–0.93)	0.93 (0.88–0.98)	40.2 (16.1–100.3)
2012 and after	9	0.79 (0.70–0.86)	0.80 (0.72–0.86)	0.86 (0.80–0.91)	14.8 (7.5–29.1)

*Se* Sensitivity, *Sp* Specificity, *AUC* Area under the HSROC curve, *DOR* Diagnostic odds ratio, *CI* Confidence interval, *FNA* Fine-needle aspiration

“—” indicates insufficient studies for pooled analysis. Two studies (Study 8 and Study 10) were excluded from the sampling time subgroup due to unique clinical contexts (FNA fluid specimen and post-intervention timing, respectively)

Robustness was tested three ways. Dropping the high-bias-risk studies (10 retained) gave pooled sensitivity 0.83 (95% CI: 0.76–0.89), specificity 0.87 (95% CI: 0.81–0.91), AUC 0.91—close to the main result. Dropping 3 studies with *n* ≤ 30 (Studies 2, 6, 8; 13 retained) gave sensitivity 0.81 (95% CI: 0.74–0.87), specificity 0.83 (95% CI: 0.77–0.88), AUC 0.89—again stable. In leave-one-out runs, sensitivity moved between 0.80 and 0.84, specificity between 0.82 and 0.86; no single study’s removal caused a big shift. Removing Study 15 (Kumbhar 2025)—the largest study but with unusually low performance (AUC 0.54)—pushed the pooled AUC to 0.91. That study’s low numbers may trace to PCT being measured at fever onset rather than during the window when necrotic infection is developing. Study 8 (Lu 2012, PCT measured in FNA aspirate fluid rather than serum) and Study 10 (Gao 2020, infection recurrence after minimally invasive treatment rather than initial IPN diagnosis) represent atypical clinical contexts that differ from the core question addressed by the remaining Group A studies. Both were retained in the primary analysis for completeness and transparency. Dropping both at once (14 retained) gave sensitivity 0.82, specificity 0.84, AUC 0.90, confirming that inclusion of these atypical studies did not materially alter the pooled estimates.

To test the subgroup difference by PCT cutoff category, we performed random-effects meta-regression using log diagnostic odds ratio as the dependent variable and cutoff group as the moderator. The moderate cutoff group included 10 studies, whereas the low cutoff group included 6 studies. The moderate cutoff group showed higher pooled diagnostic performance than the low cutoff group, with pooled DORs of 29.59 and 9.28, respectively. The global interaction test for cutoff group was statistically significant for log diagnostic odds ratio (P for interaction = 0.043), indicating a significant between-subgroup difference in overall diagnostic performance. Supplementary meta-regression showed that cutoff group was also significantly associated with logit sensitivity (P for interaction = 0.044), whereas the interaction for logit specificity was not statistically significant (P for interaction = 0.203). Descriptively, the moderate cutoff group had higher pooled sensitivity than the low cutoff group (0.85 vs. 0.72) and similar-to-higher pooled specificity (0.81 vs. 0.75). These results support the moderate cutoff range as the more informative cutoff category in the available studies, but because this comparison was based on between-study subgroup differences.

To explore whether evolving diagnostic definitions and clinical practice changes over time influenced the diagnostic performance of PCT, subgroup analysis was performed according to publication year. Studies published before 2012 showed pooled sensitivity of 0.86 (95% CI: 0.77–0.92), specificity of 0.88 (95% CI: 0.80–0.93), and HSROC AUC of 0.93 (95% CI: 0.88–0.98). Studies published from 2012 onward showed numerically lower estimates, with sensitivity of 0.79 (95% CI: 0.70–0.86), specificity of 0.80 (95% CI: 0.72–0.86), and HSROC AUC of 0.86 (95% CI: 0.80–0.91). However, HSROC meta-regression did not demonstrate a statistically significant association between publication period and diagnostic performance (*P* = 0.08).

We noted that the largest study (Kumbhar 2025; AUC 0.54) substantially affects the pooled estimate. It included 505 patients, corresponding to 23.4% of the total IPN diagnostic sample, but only 44 IPN cases. This study contributed 217 of 456 false-positive results, accounting for 47.6% of all false positives. Its individual sensitivity was 0.57, specificity was 0.53, DOR was 1.48, and AUC was 0.54. Its random-effects log-DOR model weight was 8.6%. Sensitivity analysis was performed using the same HSROC framework after excluding this study. In the full 16-study analysis, pooled sensitivity was 0.82, pooled specificity was 0.84, DOR was 23.8, and HSROC AUC was 0.90. After excluding the largest study, the corresponding estimates were 0.84, 0.82, 24.6, and 0.91, respectively (Table S5). Thus, although Kumbhar 2025 was influential because of its large sample size and disproportionate contribution to false-positive results, excluding it did not materially change the pooled diagnostic performance. We also performed an additional sensitivity analysis excluding Lu 2012 and Gao 2020, which represented atypical-context studies because Lu 2012 measured PCT in FNA aspirate fluid and Gao 2020 evaluated recurrent infection after intervention. After excluding these two studies, the analysis included 14 studies with 1,948 patients. The pooled sensitivity was 0.84, pooled specificity was 0.82, DOR was 22.7, and HSROC AUC was 0.90. These estimates were close to the main analysis, suggesting that inclusion of these two atypical-context studies did not materially affect the pooled diagnostic performance.

### PCT for predicting SAP severity

**Fig. 6:**
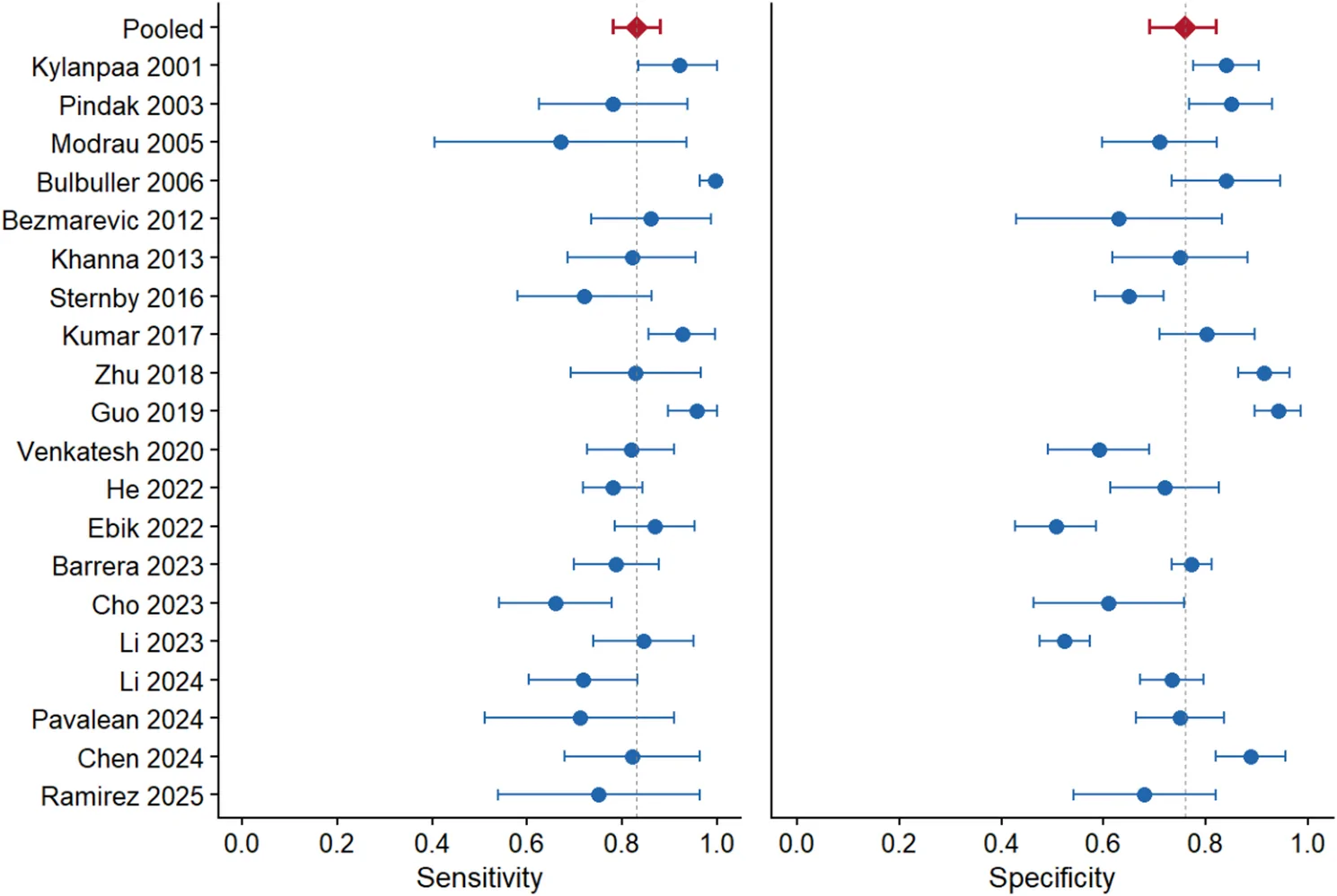
Coupled forest plots of pooled sensitivity and specificity of procalcitonin for predicting severe acute pancreatitis

**Fig. 7:**
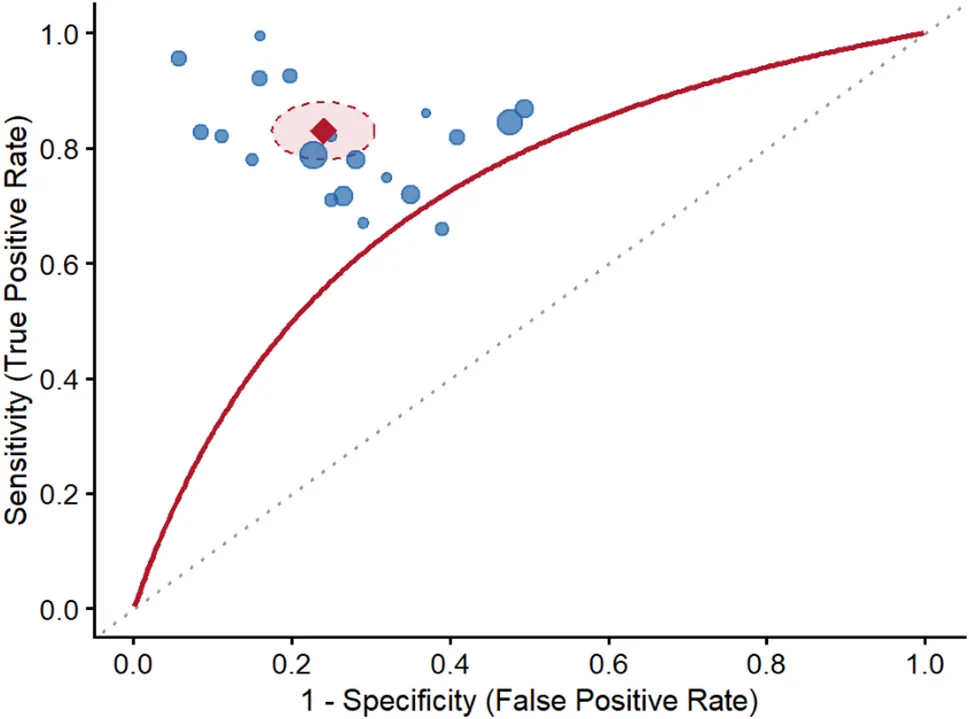
Hierarchical summary receiver operating characteristic (HSROC) curve of procalcitonin for predicting severe acute pancreatitis

The bivariate model across the 20 Group B studies produced pooled sensitivity 0.83 (95% CI: 0.78–0.88), specificity 0.76 (95% CI: 0.69–0.82), positive likelihood ratio 3.52 (95% CI: 2.68–4.62), negative likelihood ratio 0.24 (95% CI: 0.17–0.33), diagnostic odds ratio 15.7 (95% CI: 9.8–25.2), and HSROC AUC 0.87 (95% CI: 0.84–0.90). The paired forest plot and HSROC curve are in Figs. 6 and 7. Heterogeneity was high again: I² 85.3% for sensitivity and 88.7% for specificity (Q test *P* < 0.001 for both), reflecting substantial variation in patient mix, assay methods, sampling windows, and SAP definitions. The Extracted 2 × 2 contingency table of all included studies for predicting IPN and SAP is now presented in Table S6.

Stratifying by cutoff: 7 studies at ≤ 0.5 ng/mL yielded pooled sensitivity 0.87 (95% CI: 0.81–0.92), specificity 0.67 (95% CI: 0.58–0.75), AUC 0.83. Nine studies at > 0.5–<1.5 ng/mL yielded sensitivity 0.81 (95% CI: 0.74–0.87), specificity 0.79 (95% CI: 0.72–0.85), AUC 0.88. Four studies at ≥ 1.5 ng/mL yielded sensitivity 0.79 (95% CI: 0.68–0.87), specificity 0.82 (95% CI: 0.72–0.89), AUC 0.86. The expected threshold-effect pattern showed up: lower cutoffs favor sensitivity, higher cutoffs favor specificity. Stratifying by sampling time: 12 studies with a single draw at or within 24 h of admission gave sensitivity 0.81 (95% CI: 0.75–0.86), specificity 0.74 (95% CI: 0.66–0.81), AUC 0.84. The 8 with multi-timepoint follow-up sampling gave sensitivity 0.86 (95% CI: 0.79–0.91), specificity 0.79 (95% CI: 0.70–0.86), AUC 0.90—mirroring the Group A finding that serial monitoring edges out a single measurement. Stratifying by design: the 11 prospective studies had an AUC of 0.89 versus 0.84 for the 9 retrospective and other designs (Table S7). Group B included only 3 semi-quantitative PCT-Q studies, insufficient for stable bivariate model fitting; assay-method subgroup analysis was therefore not performed.

Sensitivity analyses for Group B: dropping high-bias-risk studies (12 retained) gave sensitivity 0.84 (95% CI: 0.78–0.89), specificity 0.78 (95% CI: 0.71–0.84), AUC 0.88. Dropping 2 studies with *n* ≤ 50 left the AUC at 0.87. Leave-one-out sensitivity ranged from 0.81 to 0.85, specificity from 0.73 to 0.79. Results were stable throughout.

Setting Groups A and B side by side, sensitivity was nearly identical (0.82 vs. 0.83), but specificity for IPN (0.84) clearly beat specificity for SAP (0.76). The same gap appeared in the DOR (23.8 vs. 15.7) and AUC (0.90 vs. 0.87). This makes biological sense. PCT responds preferentially to bacterial infection. When the question is “infected necrosis or sterile necrosis?“—a question defined by bacterial presence—PCT’s specificity is at its natural best. When the question is “severe or non-severe AP?“—where severity reflects a mix of factors and does not require bacterial infection—specificity drops. The SAP analysis was included as a secondary objective not to dilute the IPN focus but to provide a clinically informative contrast: the higher specificity observed for IPN than for SAP directly illustrates PCT’s preferential response to bacterial infection, reinforcing the biological rationale for its use in the IPN diagnostic pathway.

### Publication bias and evidence quality

**Fig. 8:**
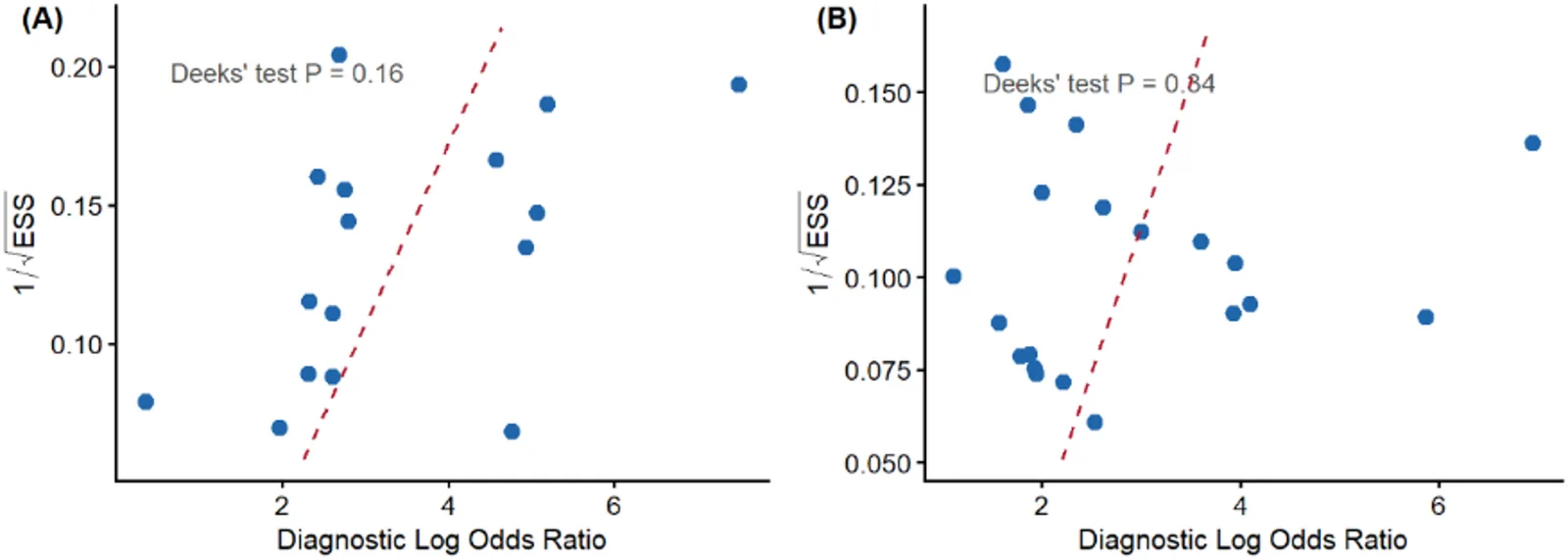
Deeks’ funnel plot asymmetry test for publication bias. **A** PCT for diagnosing IPN (*P* = 0.16). **B** PCT for predicting SAP (*P* = 0.34)

Deeks’ funnel plot results are in Fig. 8. The regression slope test gave *P* = 0.16 for Group A and *P* = 0.34 for Group B, both above the *P* < 0.10 threshold, so no clear publication bias was detected. These results deserve some caution given the limited number of studies in each pool.

Evidence certainty was graded with GRADE-DTA (Table 2). For PCT diagnosing IPN (Group A), the starting point was the high baseline that applies to cross-sectional diagnostic accuracy studies. One level was taken off for bias risk (42% of studies scored high in the index test domain because of post hoc cutoff optimization). I^2^ was high, but subgroup analyses went part of the way toward explaining it and sensitivity analyses were stable, so no downgrade for inconsistency. Indirectness, imprecision, and publication bias did not trigger downgrades. The final rating was moderate. For PCT predicting SAP (Group B), one level came off for bias risk, and a second for inconsistency-heterogeneity was high and subgroup analyses could not adequately account for it. The final rating was low.

**Table 2:** GRADE evidence profile for the diagnostic accuracy of procalcitonin in acute pancreatitis

Outcome	No. of studies	Risk of bias	Inconsistency	Indirectness	Imprecision	Publication bias	Overall certainty
PCT for diagnosing IPN	16	Serious (− 1)^a^	Serious (− 1)ᵇ	Not serious	Not serious	Not detected	⊕⊕◯◯ Low
PCT for predicting SAP	20	Serious (− 1)^a^	Serious (− 1)ᵇ	Not serious	Not serious	Not detected	⊕⊕◯◯ Low

For IPN diagnosis, heterogeneity was likely related to differences in cutoff values, sampling windows, assay platforms, and reference standards. For SAP prediction, heterogeneity was likely related to differences in cutoff values, sampling timing, SAP definitions, and detection platforms

^a^Downgraded due to the high proportion of studies with post-hoc cutoff optimization and retrospective designs

^b^Downgraded due to substantial unexplained heterogeneity in both outcomes

## Discussion

Pooling 39 studies, the 16 that examined PCT for diagnosing IPN gave an AUC of 0.90, sensitivity of 0.82, and specificity of 0.84 at low GRADE certainty. The 20 that examined PCT for predicting SAP gave an AUC of 0.87, sensitivity of 0.83, and specificity of 0.76 at low certainty. Sensitivity was similar across the two questions, but specificity was markedly higher for IPN than for SAP—in line with PCT’s preferential bacterial-infection signal. Two subgroup findings were clinically informative but should be interpreted cautiously. First, the 1.0-3.5 ng/mL cutoff subgroup showed the most favorable sensitivity-specificity balance (AUC 0.92), but this range should be viewed as a hypothesis-generating risk-stratification interval rather than a validated diagnostic threshold. Second, serial dynamic monitoring outperformed single admission testing (AUC 0.93 vs. 0.85), supporting the value of longitudinal PCT assessment in clinically suspected IPN. PCT appears to be the strongest non-invasive serum candidate for flagging IPN among markers currently in clinical use, though it cannot fully replace microbiological confirmation by FNA culture. Its most fitting role is as an aid for risk stratification and screening inside a broader decision pathway.

How do these numbers sit against earlier evidence? An older SAP-focused meta-analysis reported a pooled AUC of 0.89, close to the SAP group here, but did not break results down by cutoff or sampling time and so could not dig into heterogeneity [23]. A newer systematic review on infection prediction in acute necrotizing pancreatitis flagged a time-dependent pattern: the predictive AUC within 72 h of admission was only 0.69 and climbed to 0.86 after 72 h [24]. The sampling-time subgroup in the present work echoed that pattern from a different angle-serial monitoring AUC of 0.93 against 0.85 for single admission measurement. Two independent analyses pointing the same way lends credibility to the idea that PCT’s IPN-discriminating power is time-window dependent. A Cochrane diagnostic accuracy review, hampered by a tiny pool of high-quality studies, concluded that no single marker had enough evidence for a clinical recommendation [25]. The 16 Group A studies assembled here represent the largest PCT-for-IPN pool to date and the first to be graded under GRADE-DTA. A separate systematic review that focused on IPN risk factors found PCT to be an independent predictor of IPN in multivariable models, bolstering the case for expanding PCT from a diagnostic marker into a risk prediction tool [26].

Reference-standard heterogeneity may also have contributed to between-study variation. Studies using FNA/culture or other microbiological confirmation may show higher diagnostic performance because culture-positive IPN represents a stricter and more specific definition of infection, reducing outcome misclassification. In contrast, clinical/imaging-based criteria are more pragmatic and reflect real-world decision-making, but they may be less specific because fever, organ failure, systemic inflammation, gas on CT, extrapancreatic infection, or post-intervention changes can overlap with suspected IPN. This may partly explain why studies using FNA/culture-based definitions showed a higher AUC than those relying mainly on clinical or imaging criteria. However, this finding should not be interpreted as support for routine FNA to confirm suspected IPN. Rather, it highlights the need for standardized adjudicated reference criteria integrating clinical course, imaging, microbiological evidence when available, and procedural findings, with culture confirmation incorporated when drainage or necrosectomy is clinically performed.

The cutoff-stratified results should be interpreted with caution. In this meta-analysis, cutoffs in the 1.0–3.5 ng/mL range showed the best overall balance between sensitivity and specificity. However, these values should not be treated as fixed clinical thresholds. A PCT value below 1.0 ng/mL may lower the probability of IPN, but it cannot safely exclude IPN, especially in patients with clinical deterioration, persistent organ failure, fever, sepsis, or suspicious imaging findings. This is also supported by the pooled negative likelihood ratio of 0.22, which reduces post-test probability but does not rule out disease. Similarly, a PCT value above 1.0–3.5 ng/mL should not be used alone to diagnose IPN or to start antibiotics. It may instead serve as a warning signal that supports closer clinical reassessment. In this setting, clinicians should consider the timing of disease, serial PCT changes, CT findings, microbiological results when available, and possible extrapancreatic infections. Therefore, the 1.0–3.5 ng/mL range is better understood as a risk-stratification range rather than a stand-alone decision threshold. This interpretation is consistent with current international guidance. The revised guidelines [27, 28] state that antibiotics should be used for proven extrapancreatic infection or strong suspicion of infected necrotizing pancreatitis, but that elevated CRP, WBC count, or PCT alone should not be used to start antibiotic therapy. Thus, our findings should not be read as supporting PCT-only decision-making. Rather, they support the use of PCT as one part of a broader assessment that includes clinical course, imaging, microbiology, and evaluation for other infection sources.

Sampling time emerged as the other major performance lever. The single-admission group had an AUC of 0.85; the serial-monitoring group, 0.93. IPN is not typically present when patients walk through the door. It is a secondary infection that builds over the 2nd to 4th week of necrotizing pancreatitis. An early PCT spike is more likely to reflect the systemic inflammatory hit of pancreatitis itself rather than a bacterial signal, which puts a natural ceiling on what a one-time admission reading can do. A multicenter prospective study that tracked PCT daily for 21 days showed that sustained elevation or a secondary rise pattern carried more predictive weight than any single-time absolute value [29]. A recent review on early IPN prediction strategies made the same point about the importance of PCT trajectory and recommended routine serial monitoring for necrotizing pancreatitis patients [30]. The latest International Association of Pancreatology guidelines have adopted serial PCT monitoring as a recommended infection early-warning tool [28]. A further source of diagnostic uncertainty is that PCT reflects systemic bacterial infection rather than the anatomical source of infection. In patients with acute pancreatitis, elevated PCT may therefore arise not only from infected pancreatic necrosis but also from extrapancreatic nosocomial infections, including pneumonia, bloodstream infection, urinary tract infection, cholangitis, catheter-related infection, trauma, and organ failure, as indicated by previous publications [31, 32]. This issue is particularly relevant in patients requiring prolonged hospitalization, intensive care support, invasive procedures, or broad-spectrum antibiotics. Such infections may lead to false-positive PCT results for IPN and may partly explain the imperfect specificity and residual heterogeneity observed across studies. Accordingly, an elevated PCT value should not be interpreted as stand-alone evidence of IPN. Its clinical value is strongest when persistent elevation or a secondary rise occurs in the appropriate disease window and after alternative infectious sources have been actively considered or excluded. In practice, PCT should be integrated with clinical deterioration, contrast-enhanced CT findings, microbiological cultures where available, and careful assessment for extrapancreatic infection.

An important practical consideration not addressed by the included studies is the cost of serial PCT monitoring. The cost of a single quantitative PCT assay varies considerably across platforms and healthcare settings, and daily monitoring over a 2–3 week period in necrotizing pancreatitis could generate non-trivial cumulative costs. In high-income settings this is likely offset by potential savings from avoided unnecessary antibiotics, reduced FNA procedures, and shorter ICU stays, but formal cost-effectiveness analyses are lacking. In low- and middle-income countries, where AP-related mortality is disproportionately high, the economic burden of routine PCT monitoring may pose a significant barrier to implementation. Future studies should incorporate health-economic evaluations alongside diagnostic accuracy assessments to determine whether the clinical benefits of PCT-guided management translate into cost-effective care across different resource settings.

A related barrier is the uneven availability of PCT assays across healthcare systems. Quantitative electrochemiluminescence or immunoluminometric platforms, which formed the basis of most included studies, require laboratory infrastructure that may not be available in primary care or rural facilities. The semi-quantitative PCT-Q rapid test offers a point-of-care alternative with lower equipment requirements, but only one Group A study used this format, and its diagnostic accuracy for IPN has not been adequately validated. Establishing whether rapid semi-quantitative assays can approximate the performance of laboratory-based quantitative methods would be an important step toward broadening the clinical applicability of PCT-guided IPN management.

Separate from the diagnostic accuracy evidence for IPN, the Group C randomized trials should be interpreted as exploratory evidence for antibiotic decision support. The first, a single-center trial, found that antibiotic duration in the PCT-guided arm was significantly shorter (10.9 vs. 16.1 days) with no rise in infection-related complications [33].

The PROCAP study protocol proposed a PCT-based antibiotic algorithm [34]. In PROCAP, PCT-guided care reduced antibiotic use compared with usual care (45% vs. 63%) without an apparent safety penalty [35]. However, these findings support the potential role of PCT as an antibiotic-stewardship tool. PCT-guided antibiotic stewardship has been validated outside pancreatitis as well. A Cochrane review confirmed that the approach safely reduces antibiotic consumption in acute respiratory infections [36], and the large ICU-based PRORATA trial showed the feasibility of PCT-driven de-escalation in sepsis [37]. Taken together, these data suggest that PCT may help inform antibiotic decisions when interpreted alongside clinical course, imaging, microbiological evidence, and assessment for extrapancreatic infection, but this question remains distinct from the primary diagnostic accuracy question addressed in the present meta-analysis.

Separate from the diagnostic accuracy evidence, the Group C RCTs provided exploratory information on PCT-guided antibiotic decision support in AP. These trials suggested that PCT-guided strategies may reduce antibiotic exposure, such as shorter antibiotic duration in one single-center trial and lower antibiotic use in the PROCAP trial. However, these studies addressed antibiotic stewardship rather than the diagnostic accuracy of PCT for IPN, and they were not used to formulate stewardship recommendations in the present review. Therefore, Group C was summarized narratively and interpreted as contextual evidence only. These findings do not validate PCT as a stand-alone diagnostic test for IPN, nor do they establish a recommendation for PCT-guided antibiotic pathways in necrotizing pancreatitis.

At the molecular level, the necrotic pancreas creates a local environment—avascular pockets filled with necrotic debris and exudate—that is ideal for enteric bacteria to colonize and multiply. Once infection takes hold, the systemic response drives PCT synthesis from multiple organ sources; plasma levels should, in theory, scale with the extent of infection. A review that surveyed blood biomarkers for IPN diagnosis noted that PCT still has the strongest evidence and the highest clinical accessibility among candidates, while flagging the potential of pairing it with newer markers [38]. A separate evaluation of severity-prediction biomarkers reached the same conclusion about PCT’s central role in infection-related outcome prediction, citing its standardized assays and fast turnaround as practical advantages [39].

The high between-study heterogeneity (I^2^ > 75% for both sensitivity and specificity) warrants careful interpretation. High I^2^ values are common in diagnostic accuracy meta-analyses because even minor differences in patient spectrum, disease prevalence, threshold selection, and reference standard can shift sensitivity–specificity pairs substantially [19]. Several steps were taken to address this. First, pre-specified subgroup analyses identified PCT cutoff value and sampling time as major heterogeneity drivers: restricting to the 1.0–3.5 ng/mL cutoff range yielded more homogeneous pooled estimates (sensitivity 0.85, specificity 0.86, AUC 0.92). Second, the Spearman correlation test ruled out threshold effect as the dominant source (*r* = 0.32, *P* = 0.228). Third, multiple sensitivity analyses—removing high-bias-risk studies, small studies, and leave-one-out—all produced pooled estimates within narrow ranges of the primary results, confirming robustness. Fourth, the GRADE-DTA assessment explicitly considered inconsistency: Group A was not downgraded for this domain because subgroup analyses partially explained the variation and sensitivity analyses were stable, while Group B was downgraded one level. Despite these measures, residual heterogeneity from unstandardized assay platforms and variable reference criteria cannot be fully resolved at the meta-analytic level and remains a limitation that future primary studies should address through harmonized protocols.

This study has several limitations. The most important is the high between-study heterogeneity, which, despite extensive subgroup and sensitivity analyses, could not be fully resolved. Post hoc cutoff optimization was common across included studies. Sensitivity analysis after removing high-bias-risk papers left the pooled numbers largely unchanged, but the potential for overestimation should be kept in mind when reading the results. The IPN reference standard varied from FNA with culture to CT gas bubbles to composite clinical criteria; that inconsistency introduces disease-classification uncertainty. Next, although no language restriction was applied, dedicated Chinese bibliographic databases such as CNKI, Wanfang, VIP, and SinoMed were not searched. Therefore, language restriction and potential omission of non-English or non-indexed Chinese studies may represent sources of selection bias. Another limitation is that most included studies did not clearly separate PCT elevation caused by infected pancreatic necrosis from PCT elevation caused by infection outside the pancreas. Since PCT reflects systemic bacterial infection, it may increase in pneumonia, bloodstream infection, urinary tract infection, cholangitis, or catheter-related infection. This may have caused some false-positive results for infected pancreatic necrosis. Future studies should record extrapancreatic infections in detail and assess their effect on the diagnostic accuracy of PCT. Assay platforms differed, and differences in analytical sensitivity and linear range across platforms are a hidden heterogeneity source. Geographic coverage was uneven. Chinese studies made up more than a third of the total, while Africa and South America were almost entirely absent. AP severity and necrosis definitions changed with the 2012 Atlanta revision, and studies straddling that boundary face inherent comparability issues. A recent review of evidence-based AP management trends made the same observation: fragmented evidence and poorly standardized interventions remain ongoing obstacles for high-quality syntheses [40].

Large prospective multicenter diagnostic accuracy studies are the top priority. The 1.0-3.5 ng/mL range identified in this meta-analysis should be externally tested as a hypothesis-generating risk-stratification interval, with culture confirmation incorporated when drainage or necrosectomy is clinically performed. Head-to-head trials comparing PCT with newer infection markers-sTREM-1, presepsin, monocyte HLA-DR expression-are needed to work out the best weighting for multi-marker panels. The treatment landscape for necrotizing pancreatitis has moved from early open necrosectomy toward the minimally invasive step-up approach [41], and long-term data support the survival and outcome benefits of that shift [42]. Dynamic PCT tracking fits naturally into the step-up pathway: trends in PCT could help decide when to escalate from conservative drainage to minimally invasive debridement. A recent review on severe AP management called for biomarker-driven individualized decision models in future guidelines [43]. PCT, with the deepest evidence pool of any candidate marker, is well placed to anchor that effort.

In conclusion, pooling 16 diagnostic accuracy studies, serum PCT shows good discrimination for IPN in AP patients (AUC 0.90, pooled sensitivity 0.82, pooled specificity 0.84), graded as low certainty under the GRADE framework. Among non-invasive serum biomarkers available today, PCT is one of the best-studied biomarkers for distinguishing IPN from sterile necrosis and may be incorporated as an adjunctive biomarker for IPN risk stratification. Cutoffs in the 1.0-3.5 ng/mL range showed the best balance between sensitivity and specificity in this meta-analysis, but these values should be viewed as risk-stratification references rather than fixed clinical thresholds. Serial monitoring boosts diagnostic performance well beyond what a single admission reading can deliver. In practice, tracking PCT over time in necrotizing pancreatitis patients and watching for sustained elevation or rebound in the mid-to-late disease course matters more than any one-time number. In practice, a PCT level below 1.0 ng/mL may lower the probability of IPN, but it cannot safely exclude IPN in patients with clinical deterioration, persistent organ failure, fever, sepsis, or suspicious imaging findings. Similarly, a persistent rise or rebound of PCT above the 1.0-3.5 ng/mL range should prompt careful clinical reassessment and consideration of further workup together with disease timing, CT findings, microbiological evidence when available, and assessment for extrapancreatic infection. The heterogeneity in cutoff methods, reference definitions, and assay platforms across existing studies means that these thresholds still require validation in large prospective multicenter studies with pre-specified cutoffs and standardized, adjudicated reference criteria integrating clinical deterioration, radiologic evidence, microbiological data when available, and procedural findings, with culture confirmation incorporated when drainage or necrosectomy is clinically performed. Combining PCT with emerging infection biomarkers, validating it in adjudicated IPN diagnostic pathways, and separately evaluating its role in antibiotic decision support and step-up treatment algorithms are areas that require further study.

## Supplementary Information

Supplementary Material 1.

## Data Availability

Authors will share data upon reasonable request with researchers who provide a methodologically viable proposal and can do analyses that achieve the aims of the proposal. Data sharing requests can be directed to the corresponding author by email. A data access agreement is needed to gain access to data.

## Abbreviations

AP: Acute pancreatitis
AUC: Area under the curve
CRP: C-reactive protein
ECLIA: Electrochemiluminescence immunoassay
ELISA: Enzyme-linked immunosorbent assay
FNA: Fine-needle aspiration
HSROC: Hierarchical summary receiver operating characteristic
IPN: Infected pancreatic necrosis
PCT: Procalcitonin
SAP: Severe acute pancreatitis
SIRS: Systemic inflammatory response syndrome
