# Supplementary material for: Procalcitonin for diagnosing infected pancreatic necrosis: a systematic review and meta-analysis
Source: BMC Gastroenterol. 2026 Aug 13;26:656. doi: 10.1186/s12876-026-05216-3 (PMC13670474; doi:10.1186/s12876-026-05216-3)
Supplement: Supplementary file 1 [file 12876_2026_5216_MOESM1_ESM.docx]

1. **Supplementary Data**

**Full Search Strategy**

**PubMed/MEDLINE**

#1 "procalcitonin"[MeSH Terms]

#2 procalcitonin[Title/Abstract]

#3 pro-calcitonin[Title/Abstract]

#4 PCT[Title/Abstract]

#5 "calcitonin precursor"[Title/Abstract]

#6 #1 OR #2 OR #3 OR #4 OR #5

#7 "pancreatitis"[MeSH Terms]

#8 "pancreatitis, acute necrotizing"[MeSH Terms]

#9 "acute pancreatitis"[Title/Abstract]

#10 "necrotizing pancreatitis"[Title/Abstract]

#11 "necrotising pancreatitis"[Title/Abstract]

#12 "pancreatic necrosis"[Title/Abstract]

#13 "infected pancreatic necrosis"[Title/Abstract]

#14 "infected necrosis"[Title/Abstract]

#15 "peripancreatic necrosis"[Title/Abstract]

#16 "walled-off necrosis"[Title/Abstract]

#17 "walled off necrosis"[Title/Abstract]

#18 "pancreatic abscess"[Title/Abstract]

#19 "acute necrotizing pancreatitis"[Title/Abstract]

#20 "acute necrotising pancreatitis"[Title/Abstract]

#21 "severe acute pancreatitis"[Title/Abstract]

#22 #7 OR #8 OR #9 OR #10 OR #11 OR #12 OR #13 OR #14 OR #15 OR #16 OR #17 OR #18 OR #19 OR #20 OR #21

#23 #6 AND #22

**Embase (via Ovid)**

#1 exp procalcitonin/

#2 procalcitonin.ti,ab,kw.

#3 pro-calcitonin.ti,ab,kw.

#4 PCT.ti,ab,kw.

#5 "calcitonin precursor".ti,ab,kw.

#6 #1 OR #2 OR #3 OR #4 OR #5

#7 exp pancreatitis/

#8 exp necrotizing pancreatitis/

#9 "acute pancreatitis".ti,ab,kw.

#10 "necrotizing pancreatitis".ti,ab,kw.

#11 "necrotising pancreatitis".ti,ab,kw.

#12 "pancreatic necrosis".ti,ab,kw.

#13 "infected pancreatic necrosis".ti,ab,kw.

#14 "infected necrosis".ti,ab,kw.

#15 "peripancreatic necrosis".ti,ab,kw.

#16 "walled-off necrosis".ti,ab,kw.

#17 "walled off necrosis".ti,ab,kw.

#18 "pancreatic abscess".ti,ab,kw.

#19 "acute necrotizing pancreatitis".ti,ab,kw.

#20 "acute necrotising pancreatitis".ti,ab,kw.

#21 "severe acute pancreatitis".ti,ab,kw.

#22 #7 OR #8 OR #9 OR #10 OR #11 OR #12 OR #13 OR #14 OR #15 OR #16 OR #17 OR #18 OR #19 OR #20 OR #21

#23 #6 AND #22

#24 #23 NOT (conference abstract).pt.

Note: Line #24 excludes conference abstracts at the search stage to reduce screening burden; any potentially relevant conference records identified through reference tracking were assessed separately per the inclusion criteria.

**Cochrane Library (Cochrane Database of Systematic Reviews and Cochrane Central Register of Controlled Trials)**

#1 MeSH descriptor: [Procalcitonin] explode all trees

#2 (procalcitonin OR pro-calcitonin OR PCT OR "calcitonin precursor"):ti,ab,kw

#3 #1 OR #2

#4 MeSH descriptor: [Pancreatitis] explode all trees

#5 MeSH descriptor: [Pancreatitis, Acute Necrotizing] explode all trees

#6 ("acute pancreatitis" OR "necrotizing pancreatitis" OR "necrotising pancreatitis" OR "pancreatic necrosis" OR "infected pancreatic necrosis" OR "infected necrosis" OR "peripancreatic necrosis" OR "walled-off necrosis" OR "walled off necrosis" OR "pancreatic abscess" OR "acute necrotizing pancreatitis" OR "acute necrotising pancreatitis" OR "severe acute pancreatitis"):ti,ab,kw

#7 #4 OR #5 OR #6

#8 #3 AND #7

**Search Results Summary**

| **Database** | **Records Retrieved** |
| --- | --- |
| PubMed/MEDLINE | 187 |
| Embase (via Ovid) | 203 |
| Cochrane Library | 41 |
| **Subtotal (database search)** | **309** (after deduplication within databases) |
| Additional records from reference lists and forward citation tracking | 8 |
| **Total records identified** | **317** |
| Duplicates removed | 74 |
| Records screened (title and abstract) | 243 |

Note: The subtotal of 309 reflects that some within-database duplicates (e.g., records indexed in both CDSR and CENTRAL within Cochrane) were removed prior to export. Cross-database deduplication yielded 243 unique records for screening. Individual database hit counts (187 + 203 + 41 = 431) exceed 309 due to overlap across databases.

# 2. **Supplementary Tables**

**Table S1. Characteristics of the included studies**

***Group A: PCT for diagnosing IPN***

|  | **Study** | **Country** | **Design** | **N** | **IPN n** | **PCT Assay** | **Sampling Time** | **Cutoff (ng/mL)** | **Se (%)** | **Sp (%)** | **AUC** | **Reference Standard** |
| --- | --- | --- | --- | --- | --- | --- | --- | --- | --- | --- | --- | --- |
| 1 | (Rau et al., 1997) | Germany | Prospective | 50 | 18 | Chemoluminescent immunoassay | Daily, 14 days | 1.8 | 94 | 91 | 0.94 | FNA + culture / intraoperative culture |
| 2 | (Mándi et al., 2000) | Hungary | Prospective | 30 (10 SPN + 10 IPN + 10 sepsis) | 10 | Immunoluminometric (BRAHMS PCT Lumitest) | Daily | >1.0 | 90 | 100 | 0.97 | FNA + culture |
| 3 | (Müller et al., 2000) | Switzerland | Prospective | 64 | 12 | ELISA (Lumitest PCT, Brahms) | Daily | 1.5 | 83 | 70 | 0.79 | CECT-confirmed necrosis, IPN by clinical/culture |
| 4 | (Rau et al., 2000) | Germany | Prospective | 61 | 21 | Immunoluminometry | Daily, 14 days | 1.8 | 95 | 88 | 0.96 | FNA + culture / intraoperative culture |
| 5 | (Riché et al., 2003) | France | Prospective | 48 | 15 | Immunoluminometric (BRAHMS) | First 3 days | <2 (rule-out) | 75 | 84 | 0.81 | FNA + culture |
| 6 | (Makay et al., 2003) | Hungary | Prospective | 24 | 12 | PCT-Q (BRAHMS, semi-quantitative) | At admission | 2.0 | 75 | 83 | 0.82 | FNA + culture |
| 7 | (Rau et al., 2007) | Germany / Finland / Italy / Switzerland | Prospective, multicenter | 104 | 10 (IPN + MODS) | Semi-automated immunoassay | Daily, 21 days + weekly | 3.5 | 93 | 88 | 0.91 | Intraoperative culture |
| 8 | (Lu et al., 2012) | China | Retrospective | 30 | 18 | Chemiluminescent immunoassay | At admission | >2.0 (FNA fluid) | 94 | 92 | 0.903 | FNA + culture |
| 9 | (Chen et al., 2017) | China | Retrospective | 215 | 87 | ECLIA (Roche) | Within 48 h | 0.5 | 67.8 | 77.3 | 0.674 | FNA + culture; Atlanta classification |
| 10 | (Gao et al., 2020) | China | Retrospective | 178 | 13 (infection recurrence) | ECLIA (Roche) | At catheter removal | 0.05 | 77 | 83 | 0.78 | FNA; Atlanta classification |
| 11 | (Alberti et al., 2022) | Spain | Prospective | 152 | 22 | ECLIA | Admission (first 72 h) | 0.68 | 80 | 72 | 0.79 | Blood culture; Atlanta classification |
| 12 | (Jakkampudi et al., 2023) | India | Prospective | 56 (Cohort-1) + 78 (Cohort-2) | 26 + 57 | ELISA | At admission | 1.2 | 78 | 74 | 0.80 | FNA + culture |
| 13 | (He et al., 2023) | China | Retrospective | 149 (SAP) | 47 | ECLIA (Roche) | Within 48 h | 1.5 | 76 | 81 | 0.82 | Culture + imaging |
| 14 | (Zhu et al., 2023) | China | Retrospective | 324 (SAP) | 68 | ECLIA (Roche) | Day 1, 4, 7 | 1.8 | 97.2 (combined) | 77.2 (combined) | 0.92 (combined) | FNA; Atlanta classification; CTSI |
| 15 | (Kumbhar et al., 2025) | India | Prospective | 505 | 44 | ECLIA | At fever onset | 0.8 | 56 | 53 | 0.54 | Culture |
| 16 | (Zhang et al., 2025) | China | Retrospective | 88 | 32 | ECLIA (Roche) | At admission | 1.0 | 81 | 76 | 0.78 | FNA; Atlanta classification |

***Group B: PCT for predicting SAP***

|  | **Study** | **Country** | **Design** | **N** | **SAP n** | **PCT Assay** | **Sampling Time** | **Cutoff (ng/mL)** | **Se (%)** | **Sp (%)** | **AUC** |
| --- | --- | --- | --- | --- | --- | --- | --- | --- | --- | --- | --- |
| a17 | (Kylänpää-Bäck et al., 2001) | Finland | Prospective | 162 | 38 | PCT-Q (semi-quantitative) | Admission + 24 h | 0.5 | 92 (24 h) | 84 (24 h) | 0.90 |
| 18 | (Pindak et al., 2003) | Slovakia | Prospective | 101 | 27 | Immunoluminometric (BRAHMS) | Admission + 12 h | 5.0 (mortality) | 78 | 85 | 0.84 |
| 19 | (Modrau et al., 2005) | Denmark | Prospective | 75 | 12 | Immunoluminometric (BRAHMS) | Admission + 48 h | 2.0 | 67 | 71 | 0.61 (adm), 0.71 (48 h) |
| 20 | (Bülbüller et al., 2006) | Turkey | Prospective | 65 | 19 | ELISA | Admission + follow-up | 0.5 | 100 | 84 | 0.93 |
| 21 | (Bezmarević et al., 2012) | Serbia | Prospective | 51 | 29 | BRAHMS Kryptor | 24 h | 0.5 | 86 | 63 | 0.78 |
| 22 | (Khanna et al., 2013) | India | Prospective | 72 | 31 | PCT-Q (semi-quantitative) | At admission | 0.5 | 82 | 75 | 0.81 |
| 23 | (Sternby et al., 2016) | Sweden | Prospective | 232 | 39 | ELISA | Admission | Preset | 72 | 65 | 0.68 |
| 24 | (Kumar et al., 2017) | Nepal | Prospective | 125 | 54 | ECLIA | 24-72 h | 0.99 | 92.6 | 80.3 | 0.887 |
| 25 | (Zhu et al., 2018) | China | Retrospective | 146 (elderly) | 29 | ECLIA (Roche) | At admission | 1.5 | 82.8 | 91.5 | 0.908 |
| 26 | (Guo et al., 2019) | China | Retrospective | 149 (SAP) | 45 (IFD) | ECLIA (Roche) | Within 4 h | 2.0 | 95.6 | 94.2 | 0.97 |
| 27 | (Venkatesh et al., 2020) | India | Prospective, observational | 164 | 69 (CT) | ECLIA | Admission | 0.5 | 81.8 | 59.1 | 0.771 |
| 28 | (He et al., 2022) | China | Retrospective | 238 | 170 (MSAP + SAP) | ECLIA (Roche) | Within 48 h | 1.0 | 78 | 72 | 0.853 (combined) |
| 29 | (Ebik et al., 2022) | Turkey | Cross-sectional | 214 | 62 | ECLIA | First 12 h | 0.94 | 86.9 | 50.7 | 0.731 |
| 30 | (Barrera Gutierrez et al., 2023) | USA | Prospective registry | 516 | 80 | ECLIA | Baseline | 0.155 | 78.7 | 77.3 | 0.796 |
| 31 | (Cho et al., 2023) | South Korea | Retrospective | 103 | 42 mild / 61 non-mild | Cobas (Roche) | Admission | 0.5 | 66 | 61 | 0.62 |
| 32 | (Li et al., 2023) | China | Retrospective | 436 | 45 | ECLIA (Roche) | Within 24 h | 1.2 | 84.4 | 52.4 | 0.75 |
| 33 | (Li et al., 2024) | China | Retrospective | 253 | 60 | ECLIA (Roche) | At admission | 0.8 | 71.7 | 73.4 | 0.795 |
| 34 | (Pavalean et al., 2024) | Romania | Retrospective | 116 | 20 | ECLIA | First 24 h | 1.0 | 71 | 75 | 0.753 |
| 35 | (Chen et al., 2024) | China | Prospective | 109 | 28 | ELISA | At admission | 1.5 | 82 | 88.9 | 0.83 |
| 36 | (Ramirez-Gonzalez et al., 2025) | Mexico | Observational | 59 (SAP) | 16 (deaths) | ELISA | Day 1 + Day 3 | 1.26 (72 h, mortality) | 75 | 68 | 0.70 |

***Group C: PCT-guided antibiotic therapy***

|  | **Study** | **Country** | **Design** | **N** | **PCT Cutoff (ng/mL)** | **Main Finding** |
| --- | --- | --- | --- | --- | --- | --- |
| 37 | (Qu et al., 2012) | China | RCT, single-center | 71 (35 vs 36) | 0.5 | PCT-guided group had significantly shorter antibiotic duration (10.9 vs 16.1 days) and shorter hospital stay |
| 38 | (Siriwardena et al., 2019) | UK | RCT protocol | 200 (planned) | 1.0 | Protocol paper, no outcome data |
| 39 | (Siriwardena et al., 2022) | UK | RCT, single-center, patient-blinded | 260 (132 vs 128) | 1.0 | PCT-guided group had lower antibiotic use (45% vs 63%) with no adverse effects |

*Note: Abbreviations: AP, acute pancreatitis; AUC, area under the curve; CECT, contrast-enhanced computed tomography; ECLIA, electrochemiluminescence immunoassay; ELISA, enzyme-linked immunosorbent assay; FNA, fine-needle aspiration; IFD, intestine functional disturbance; IPN, infected pancreatic necrosis; MODS, multi-organ dysfunction syndrome; MSAP, moderately severe acute pancreatitis; PCT, procalcitonin; SAP, severe acute pancreatitis; Se, sensitivity; Sp, specificity; SPN, sterile pancreatic necrosis.*

**Table S2. Standardized Data Extraction Template**

| **Field** | **Description** | **Options / Format** |
| --- | --- | --- |
| **Study Characteristics** | | |
| Study ID | Sequential number assigned to each study | Numeric |
| First author | Last name of first author | Text |
| Publication year | Year of publication | YYYY |
| Country | Country where study was conducted | Text |
| Study design | Type of study design | Prospective / Retrospective / Cross-sectional / RCT |
| Single or multicenter | Number of participating centers | Single / Multicenter |
| **Patient Characteristics** | | |
| Total sample size (N) | Number of patients enrolled | Numeric |
| Age, mean or median | Central tendency of patient age | Numeric (± SD or IQR) |
| Male sex, n (%) | Number and proportion of male patients | Numeric (%) |
| AP etiology | Predominant etiology of acute pancreatitis | Biliary / Alcoholic / Hypertriglyceridemia / Mixed / Not reported |
| AP severity distribution | Proportion of mild, moderately severe, and severe AP | MAP n (%) / MSAP n (%) / SAP n (%) |
| IPN cases, n (%) | Number and proportion of patients with infected pancreatic necrosis (Group A) | Numeric (%) |
| SAP cases, n (%) | Number and proportion of patients classified as severe AP (Group B) | Numeric (%) |
| **PCT Test Details** | | |
| PCT assay method | Commercial platform or method used for PCT measurement | ECLIA / ELISA / Immunoluminometric / PCT-Q (semi-quantitative) / Other |
| Assay manufacturer | Manufacturer and model of the assay kit | Text |
| Sampling time | Timing of blood draw relative to admission or disease onset | At admission / Within 24 h / Within 48 h / Within 72 h / Daily serial / Multiple time points / Other |
| PCT cutoff value (ng/mL) | Diagnostic threshold used to classify PCT-positive and PCT-negative | Numeric |
| Cutoff determination method | How the cutoff was selected | Pre-specified / ROC curve optimization (post hoc) / Literature-based / Manufacturer recommendation / Not reported |
| **Reference Standard** | | |
| IPN reference standard (Group A) | Method used to confirm or exclude IPN | FNA + microbiological culture / Intraoperative tissue culture / CECT gas bubbles + clinical evidence / Composite clinical-radiological judgment |
| SAP reference standard (Group B) | Severity grading system used | Revised Atlanta Classification / Atlanta 1992 / APACHE II / Ranson / Other |
| **Diagnostic Classification Data** | | |
| True positives (TP) | Number of patients correctly identified as positive | Numeric |
| False positives (FP) | Number of patients incorrectly identified as positive | Numeric |
| False negatives (FN) | Number of patients incorrectly identified as negative | Numeric |
| True negatives (TN) | Number of patients correctly identified as negative | Numeric |
| Sensitivity (Se) | TP / (TP + FN) | Numeric (%) |
| Specificity (Sp) | TN / (TN + FP) | Numeric (%) |
| AUC | Area under the ROC curve, if reported | Numeric |
| **Data Derivation Notes** | | |
| Were 2×2 data directly reported? | Whether TP/FP/FN/TN were explicitly provided | Yes / No (back-calculated from Se, Sp, and N) |
| Multiple cutoffs reported? | Whether the study reported more than one cutoff | Yes (list all) / No |
| Cutoff used in primary analysis | Which cutoff was selected for the main pooled analysis | Author-labeled optimal / Most commonly used in the field |

**Table S3. Tailored QUADAS-2 Signaling Questions and Judgment Criteria**

*Domain 1: Patient Selection*

| **Signaling Question** | **Judgment Criteria** |
| --- | --- |
| Was a consecutive or random sample of patients enrolled? | Low risk: Consecutive enrollment or prospective cohort with pre-defined inclusion criteria. High risk: Convenience sampling, retrospective chart review with unclear selection, or enrollment restricted to specific severity subgroups only. Unclear: Insufficient information on enrollment method. |
| Was a case-control design avoided? | Low risk: Cross-sectional or cohort design in which all AP patients were eligible. High risk: Separate recruitment of IPN/SAP cases and controls. Unclear: Not described. |
| Did the study avoid inappropriate exclusions? | Low risk: No exclusion of patients who might have affected diagnostic accuracy estimates (e.g., patients with indeterminate results were retained). High risk: Exclusion of patients with borderline PCT values, mild AP only populations, or exclusion of patients who did not receive the reference standard. Unclear: Exclusion criteria not fully described. |
| **Applicability**: Are there concerns that included patients do not match the review question? | Low concern: Unselected adult AP patients regardless of severity. High concern: Highly selected population (e.g., ICU-only or post-intervention cohort). |

*Domain 2: Index Test (PCT)*

| **Signaling Question** | **Judgment Criteria** |
| --- | --- |
| Were the index test results interpreted without knowledge of the reference standard results? | Low risk: PCT measurement performed and interpreted before or independently of the IPN/SAP determination. High risk: PCT result known to clinicians who also determined the reference standard outcome. Unclear: Blinding not described. |
| If a threshold was used, was it pre-specified? | Low risk: PCT cutoff was pre-defined before data analysis, adopted from prior literature, or based on manufacturer recommendation. High risk: Cutoff was selected post hoc by optimizing the ROC curve on the study's own data. Unclear: Cutoff determination method not reported. |
| **Applicability**: Are there concerns that the index test, its conduct, or interpretation differ from the review question? | Low concern: Quantitative PCT assay with a defined numeric cutoff. High concern: Semi-quantitative PCT-Q with categorical bands rather than continuous values. |

*Domain 3: Reference Standard*

| **Signaling Question** | **Judgment Criteria** |
| --- | --- |
| Is the reference standard likely to correctly classify the target condition? | Low risk (Group A): FNA with positive microbiological culture, or intraoperative tissue culture with microbiological confirmation. Low risk (Group B): Revised Atlanta Classification 2012 applied with full clinical, laboratory, and imaging data. High risk (Group A): IPN determined solely by CT gas bubbles without microbiological proof, or clinical judgment alone. High risk (Group B): Severity classification based on outdated or non-standardized scoring systems without clear criteria. Unclear: Reference standard described but criteria for positive/negative not fully specified. |
| Were the reference standard results interpreted without knowledge of the index test results? | Low risk: Reference standard determination was independent of PCT results. High risk: Knowledge of PCT results influenced decisions to perform FNA or to classify patients. Unclear: Not described. |
| **Applicability**: Are there concerns that the target condition as defined by the reference standard does not match the review question? | Low concern (Group A): Microbiologically confirmed IPN. High concern (Group A): IPN defined by imaging features alone or by composite clinical criteria without culture confirmation, introducing potential misclassification. |

*Domain 4: Flow and Timing*

| **Signaling Question** | **Judgment Criteria** |
| --- | --- |
| Was there an appropriate interval between the index test and reference standard? | Low risk: PCT measurement and reference standard determination occurred within the same hospitalization episode, with the reference standard applied at the clinically appropriate time point for IPN/SAP ascertainment. High risk: Interval between PCT sampling and outcome determination exceeded 4 weeks, or PCT was measured at a time point clinically disconnected from the outcome (e.g., PCT at admission but IPN assessed only at surgery weeks later without interim monitoring). Unclear: Timing not reported. |
| Did all patients receive the same reference standard? | Low risk: All enrolled patients underwent the same reference standard procedure. High risk: Only patients with clinical suspicion of IPN received FNA (partial verification bias); patients without suspicion were classified as sterile necrosis without microbiological confirmation. Unclear: Not described. |
| Were all patients included in the analysis? | Low risk: All enrolled patients accounted for in the 2×2 table; no unexplained exclusions after enrollment. High risk: Patients excluded after enrollment due to missing PCT data, indeterminate results, or loss to follow-up without explanation. Unclear: Flow diagram absent or incomplete. |

**Table S4. Post-test probability of IPN for IPN using the hypothesis-generating PCT cutoff of 1-3.5 ng/mL**

| Assumed pre-test probability of IPN | Post-test probability after positive PCT | Post-test probability after negative PCT |
| --- | --- | --- |
| 10% | 40.3% | 1.9% |
| 20% | 60.3% | 4.2% |
| 30% | 72.2% | 7.0% |
| 40% | 80.2% | 10.4% |
| 50% | 85.9% | 14.9% |

Note: IPN, infected pancreatic necrosis; PCT, procalcitonin. Post-test probabilities were calculated using likelihood ratios derived from the pooled sensitivity and specificity of the >1.0–≤3.5 ng/mL PCT cutoff subgroup. For this subgroup, the pooled sensitivity was 0.85 and the pooled specificity was 0.86, corresponding to a positive likelihood ratio of 6.07 and a negative likelihood ratio of 0.17.

**Table S5 Sensitivity analysis of meta-analysis for IPN diagnosis excluding the study with the largest sample size**

| **Analysis** | **No. of studies** | **Pooled sen** | **Pooled spe** | **DOR** | **AUC** |
| --- | --- | --- | --- | --- | --- |
| **All** | **16** | **0.82 (0.75–0.87)** | **0.84 (0.78–0.89)** | **23.8 (12.6–44.9)** | **0.90**  **(0.86-0.94)** |
| **Excluding Kumbhar 2025** | **15** | **0.84 (0.79-0.89)** | **0.82 (0.77-0.86)** | **24.6 (14.6-41.6)** | **0.91 (0.87-0.94)** |
| **Kumbhar 2025 alone** | **1** | **0.57** | **0.53** | **1.48** | **0.54** |
|  | **Total sample contribution** | **IPN case contribution** | **Non-IPN control contribution** | **False-positive results contributed** | **Random effects log-DOR model weight** |
| **Kumbhar 2025 alone** | **23.4% (505/2156)** | **8.6 % (44/512)** | **28.0% (461/1644)** | **47.6% (217/456)** | **8.6%** |

Se, sensitivity; Sp, specificity; AUC, area under the HSROC curve; DOR, diagnostic odds ratio; IPN, Infected Pancreatic Necrosis.

**Table S6 Extracted 2×2 contingency tables of all included studies for diagnostic meta-analysis**

| Study ID | TP | FP | FN | TN |
| --- | --- | --- | --- | --- |
| PCT for predicting IPN | | | | |
| Rau 1997 | 17 | 3 | 1 | 29 |
| Mándi 2000 | 9 | 0 | 1 | 20 |
| Müller 2000 | 10 | 16 | 2 | 36 |
| Rau 2000 | 20 | 5 | 1 | 35 |
| Riché 2003 | 11 | 5 | 4 | 28 |
| Makay 2003 | 9 | 2 | 3 | 10 |
| Rau 2007 | 9 | 11 | 1 | 83 |
| Lu 2012 | 17 | 1 | 1 | 11 |
| Chen 2017 | 59 | 29 | 28 | 99 |
| Gao 2020 | 10 | 28 | 3 | 137 |
| Alberti 2022 | 18 | 36 | 4 | 94 |
| Jakkampudi 2023 | 65 | 13 | 18 | 38 |
| He 2023 | 36 | 19 | 11 | 83 |
| Zhu 2023 | 66 | 58 | 2 | 198 |
| Kumbhar 2025 | 25 | 217 | 19 | 244 |
| Zhang 2025 | 26 | 13 | 6 | 43 |
| PCT for predicting SAP | | | | |
| Kylänpää 2001 | 35 | 20 | 3 | 104 |
| Pindak 2003 | 21 | 11 | 6 | 63 |
| Modrau 2005 | 8 | 18 | 4 | 45 |
| Bülbüller 2006 | 19 | 7 | 0 | 39 |
| Bezmarević 2012 | 25 | 8 | 4 | 14 |
| Khanna 2013 | 25 | 10 | 6 | 31 |
| Sternby 2016 | 28 | 68 | 11 | 125 |
| Kumar 2017 | 50 | 14 | 4 | 57 |
| Zhu 2018 | 24 | 10 | 5 | 107 |
| Guo 2019 | 43 | 6 | 2 | 98 |
| Venkatesh 2020 | 56 | 39 | 13 | 56 |
| He 2022 | 133 | 19 | 37 | 49 |
| Ebik 2022 | 54 | 75 | 8 | 77 |
| Barrera 2023 | 63 | 99 | 17 | 337 |
| Cho 2023 | 40 | 16 | 21 | 26 |
| Li 2023 | 38 | 186 | 7 | 205 |
| Li 2024 | 43 | 51 | 17 | 142 |
| Pavalean 2024 | 14 | 24 | 6 | 72 |
| Chen 2024 | 23 | 9 | 5 | 72 |
| Ramirez 2025 | 12 | 14 | 4 | 29 |

Note: TP, true positive; FP, false positive; FN, false negative; TN, true negative; IPN, Infected Pancreatic Necrosis; SAP, severe acute pancreatitis.

**Table S7. Subgroup Analyses of the Diagnostic Accuracy of Procalcitonin for Predicting Severe Acute Pancreatitis (Group B)**

| **Subgroup** | **No. of studies** | **Pooled Se (95% CI)** | **Pooled Sp (95% CI)** | **AUC** |
| --- | --- | --- | --- | --- |
| **Overall** | 20 | 0.83 (0.78–0.88) | 0.76 (0.69–0.82) | 0.87 (0.84-0.90) |
| **By PCT cutoff** |  |  |  |  |
| ≤0.5 ng/mL | 7 | 0.87 (0.81–0.92) | 0.67 (0.58–0.75) | 0.83 (0.78-0.88) |
| >0.5–≤1.5 ng/mL | 9 | 0.81 (0.74–0.87) | 0.79 (0.72–0.85) | 0.88 (0.85-0.92) |
| >1.5 ng/mL | 4 | 0.79 (0.68–0.87) | 0.82 (0.72–0.89) | 0.86 (0.81-0.91) |
| **By sampling time** |  |  |  |  |
| Admission / early single (≤24 h) | 12 | 0.81 (0.75–0.86) | 0.74 (0.66–0.81) | 0.84 (0.81-0.86) |
| Multi-timepoint / follow-up | 8 | 0.86 (0.79–0.91) | 0.79 (0.70–0.86) | 0.90 (0.84-0.93) |
| **By study design** |  |  |  |  |
| Prospective | 11 | — | — | 0.89 (0.85-0.93) |
| Retrospective and other | 9 | — | — | 0.84 (0.81-0.87) |

Note: Se, sensitivity; Sp, specificity; AUC, area under the HSROC curve; CI, confidence interval. "—" indicates that only AUC was reported for this subgroup in the primary analysis. Group B included only 3 semi-quantitative PCT-Q studies, insufficient for stable bivariate model fitting; assay-method subgroup analysis was therefore not performed.
